# Computational modelling reveals contrasting effects on reinforcement learning and cognitive flexibility in stimulant use disorder and obsessive-compulsive disorder: remediating effects of dopaminergic D2/3 receptor agents

**DOI:** 10.1007/s00213-019-05325-w

**Published:** 2019-07-20

**Authors:** Jonathan W. Kanen, Karen D. Ersche, Naomi A. Fineberg, Trevor W. Robbins, Rudolf N. Cardinal

**Affiliations:** 1 Department of Psychology, University of Cambridge, Cambridge, UK; 2 Behavioural and Clinical Neuroscience Institute, University of Cambridge, Cambridge, UK; 3 Department of Psychiatry, University of Cambridge, Cambridge, UK; 4 Hertfordshire Partnership University NHS Foundation Trust, Welwyn Garden City, Hertfordshire, UK; 5 Department of Postgraduate Medicine, College Lane Hatfield, University of Hertfordshire, Hertfordshire, UK; 6 Cambridgeshire and Peterborough NHS Foundation Trust, Cambridge, UK

**Keywords:** Obsessive-compulsive disorder, Stimulant use disorder, Addiction, Compulsivity, Reversal learning, Reinforcement learning, Computational modelling, Dopamine, Amisulpride, Pramipexole

## Abstract

**Rationale:**

Disorders of compulsivity such as stimulant use disorder (SUD) and obsessive-compulsive disorder (OCD) are characterised by deficits in behavioural flexibility, some of which have been captured using probabilistic reversal learning (PRL) paradigms.

**Objectives:**

This study used computational modelling to characterise the reinforcement learning processes underlying patterns of PRL behaviour observed in SUD and OCD and to show how the dopamine D_2/3_ receptor agonist pramipexole and the D_2/3_ antagonist amisulpride affected these responses.

**Methods:**

We applied a hierarchical Bayesian method to PRL data across three groups: individuals with SUD, OCD, and healthy controls. Participants completed three sessions where they received placebo, pramipexole, and amisulpride, in a double-blind placebo-controlled, randomised design. We compared seven models using a bridge sampling estimate of the marginal likelihood.

**Results:**

Stimulus-bound perseveration, a measure of the degree to which participants responded to the same stimulus as before irrespective of outcome, was significantly increased in SUD, but decreased in OCD, compared to controls (on placebo). Individuals with SUD also exhibited reduced reward-driven learning, whilst both the SUD and OCD groups showed increased learning from punishment (nonreward). Pramipexole and amisulpride had similar effects on the control and OCD groups; both increased punishment-driven learning. These D_2/3_-modulating drugs affected the SUD group differently, remediating reward-driven learning and reducing aspects of perseverative behaviour, amongst other effects.

**Conclusions:**

We provide a parsimonious computational account of how perseverative tendencies and reward- and punishment-driven learning differentially contribute to PRL in SUD and OCD. D_2/3_ agents modulated these processes and remediated deficits in SUD in particular, which may inform therapeutic effects.

**Electronic supplementary material:**

The online version of this article (10.1007/s00213-019-05325-w) contains supplementary material, which is available to authorized users.

## Introduction

Optimal functioning and wellbeing requires flexible adaptation of behaviour to maximise rewards and minimise punishments. Many psychiatric disorders involve aberrant processing of, and responding to, rewarding and aversive experiences. Compulsivity is a hallmark of stimulant use disorder (SUD) and obsessive-compulsive disorder (OCD), where behaviour to obtain reward or avoid punishment, inappropriately persists, resulting in undesirable consequences. In SUD, drug-taking habits prevail despite the risk of family breakup or job loss. Individuals with OCD are unable to desist repetitive behaviours, which can consume large amounts of time and ultimately compromise social or occupational functioning (APA 2013).

Deficits in behavioural flexibility can be captured in a laboratory setting using probabilistic reversal learning (PRL) paradigms (Lawrence et al. 1999). Adaptive behaviour involves a trade-off between flexibly updating actions when the environment changes and ignoring rare events when the environment is stable. PRL models this trade-off. Participants are presented with two choices and learn by trial and error which option is correct most of the time. Ignoring spurious minority feedback leads to more rewards overall, and is thus adaptive. The contingencies are then reversed, and participants must update their choices to maximise rewards again. In these experiments, analysed using classical statistics, individuals with SUD show perseverative deficits—impairments in the ability to update behaviour when circumstances change (Ersche et al. 2008, 2011). Whilst patients with OCD also exhibit behavioural inflexibility, the most consistent evidence comes from the extra-dimensional shifting paradigm, which requires shifting attention from one aspect of a compound stimulus to another, to maximise reinforcement (Chamberlain et al. 2007a). The findings on PRL in OCD, on the other hand, are mixed (Chamberlain et al. 2007b; Ersche et al. 2011; Remijnse et al. 2006). At the same time, individuals with depression (Murphy et al. 2003; Taylor Tavares et al. 2008) instead show hypersensitivity to spurious negative feedback in PRL, manifested by inappropriately changing behaviour following punishment when it is rare. To our knowledge, however, nobody has compared the microstructure of behaviour in PRL between disorders of compulsivity using computational models of reinforcement learning (RL).

Techniques for analysing behaviour that are based on RL describe the behaviour in question—for instance, choice—by having a computer simulate putative psychological processes, such as learning from reward or punishment, tending to choose recently chosen stimuli or respond to recently responded-to locations irrespective of outcome, and selecting between alternative actions. These computational processes are governed by parameters (e.g. a given subject’s tendency to learn from reward, or from aversive feedback such as errors). In turn, those parameters may be influenced by dynamic pharmacological manipulations and may vary according to relatively static properties of the subject, such as psychiatric disorders. The most likely values for those parameters are discovered by fitting the predictions of a computational RL model to actual behaviour. In the most informative kind of analysis (Daw 2011), a Bayesian hierarchy is used. For example, subjects are drawn from groups and are influenced by drug manipulations, so the parameters pertaining to a given subject in a given condition (or session) exist “beneath” the level of groups and drugs; at the lowest level, trial-by-trial data are predicted and compared to behaviour. Finally, the best RL model may be selected from a number of competing alternatives according to a formal Bayesian procedure, penalizing models that fit badly or that are over-complex (Occam’s razor). Analysing behavioural data using a hierarchical Bayesian RL approach therefore simultaneously allows the best computational model of behaviour to be selected from candidate models—allowing psychological processes to be inferred—and the parameters of that model to be characterized, to uncover the effects of disorders or pharmacological manipulations on those processes.

Here, we took a transdiagnostic approach to interrogate the computational processes underlying maladaptive behaviour across two disorders of compulsivity: SUD and OCD. We applied RL models in a reanalysis of behavioural data on PRL from Ersche et al. (2011), which enabled a direct comparison of these groups. The original study by Ersche et al. (2011) also investigated the effects of the dopaminergic D_2/3_ receptor agonist pramipexole and the D_2/3_ antagonist amisulpride. Using classical statistics, they showed pramipexole remediated perseverative behaviour in SUD and normalised the corresponding hypoactivity in the head of the caudate; however, their analysis did not detect any further effects of pramipexole or amisulpride in SUD, OCD, or controls. We additionally sought to deconstruct the influence of dopaminergic agents on computational processes underlying PRL in these groups. Understanding D_2/3_ receptor involvement in maladaptive behaviour is particularly important given the evidence of reduced striatal D_2_ receptor availability in cocaine abuse (Volkow et al. 1993), methamphetamine abuse (Volkow et al. 2001), and OCD (Denys et al. 2004; Perani et al. 2008; but see Schneier et al. 2008). D_2/3_ antagonists, additionally, are effective in augmenting first-line selective serotonin reuptake inhibitor (SSRI) therapy in treatment-resistant cases of OCD (Fineberg et al. 2013).

The primary aim of our modelling approach was to deepen our understanding of how SUD and OCD differ and overlap, and to do so more robustly and with greater detail than the conventional analyses previously reported. Using data from Ersche et al. (2011), we asked whether behavioural differences could be best accounted for by algorithms describing how rewarding and punishing outcomes drive action, for instance, or if models incorporating additional elements tracking behavioural tendencies independent of action-outcome contingencies—“stickiness” parameters—would yield more optimal characterisations. Experimental data showing abnormalities in processing and flexibility adapting behaviour following rewards and punishments in SUD (e.g. Ersche et al. 2011, 2016) and OCD (e.g. Gillan et al. 2011, 2014) suggest parameters tracking separate reward and punishment learning rates would be of central importance. We predicted separate learning rates, for positive and negative outcomes, would be superior to a single reinforcement rate, and could enable the detection of asymmetries in appetitive and aversive processing—avoiding negative consequences is a key feature of OCD (APA 2013), and is not central in SUD, for instance. At the same time, because compulsivity may stem from maladaptive stimulus-response habits, where behaviour persists irrespective of outcome (Everitt and Robbins 2016; Gillan et al. 2011, 2014), we expected the addition of stickiness parameters would be optimal. Finally, we asked whether our data would instead be better characterised by a different model, used to dissect perseverative behaviour (den Ouden et al. 2013), that tests the balance of how incoming information is valued against current beliefs (based on past experience). We expected that analysing behaviour in this more sophisticated manner would enable us to better differentiate the SUD and OCD groups and characterise their response to dopaminergic agents.

## Methods

### Participants

The study included 56 participants, composed of 19 healthy volunteers, 18 patients with SUD, and 19 patients with OCD. Diagnoses of stimulant dependence and OCD were ascertained using the structured clinical interview for the DSM-IV (First et al. 2002). Here, we use the term substance use disorder (SUD), which is the current nomenclature in the DSM-V (APA 2013), rather than stimulant dependence, as used in the DSM-IV-TR (APA 2000). Within the SUD group, 10 participants met DSM-IV-TR (APA 2000) criteria for cocaine/crack dependence while 8 met criteria for amphetamine dependence. Individuals with SUD had a history of illicit stimulant dependence for a minimum of 2 years. Participants did not have any other Axis I psychiatric disorder at the time of the study and were not taking any other medication aside from SSRIs in the OCD group. Both the SUD and OCD groups, however, had elevated depressive symptoms, which is reported in the “Results” section. Use of illicit drugs, besides in the SUD group, was an exclusion criterion. Participants were assessed for their general health, which included a physical examination and clinical blood tests at baseline, and were excluded if they had a history of any serious medical condition. The study was approved by the Cambridge Research Ethics Committee and all participants provided written informed consent. Further information on the three groups of participants, including their demographics, baseline personality measures, and clinical information are presented in Table 1.

**Table 1:** Demographic, psychological and baseline personality measures for the groups of healthy controls (HC; *n* = 18), individuals with stimulant use disorder (SUD; *n* = 17), and individuals with obsessive-compulsive disorder (OCD; n = 18). Mean (standard deviation)

Group	HC	SUD	OCD	*F*	*df*	*P*
Age (years)	32.7 (± 6.9)	34.3 (± 7.4)	35.4 (± 9.8)	0.49	2.50	0.618
Gender ratio (male/female)	15:3	14:3	7:11			0.318^a^
Ethnic ratio (Caucasian:Afro-Caribbean)	17:1	15:2	18:00			0.308^a^
Verbal intelligence quotient (NART)	108.4 (± 6.0)	108.0 (± 8.3)	107.9 (± 8.8)	0.06	2.50	0.938
Years of education	12.4 (± 1.8)	11.2 (± 1.0)	12.3 (± 2.0)	2.06	2.50	0.082
Dysphoric mood, BDI-II (total score at baseline)	1.1 (± 2.4)	9.8 (± 11.2)	18.5 (± 10.0)	18.07	2.50	< 0.001
Impulsivity, BIS-11 (total score)	62.0 (± 7.2)	81.7 (± 9.7)	66.9 (± 9.7)	22.83	2.49	< 0.001
Compulsivity, Y-BOCS (total score)	0.1 (± 0.5)	–	24.11 (± 13.0)	–	–	–
Compulsivity, OCDUS (total score)	–	26.0 (± 7.8)	–	–	–	–
Age of onset (years) of stimulant abuse or of OCD	–	20.5 (± 5.4)	17.1 (± 11.0)	–	–	–
Duration (years) of stimulant abuse or of OCD	–	11.7 (± 7.4)	18.3 (± 10.6)	–	–	–

Reproduced with permission from Ersche et al. (2011)

*NART* National Adult Reading Test, *BDI-II* Beck Depression Inventory, version II (Beck et al. 1996), *BIS-11* Barratt Impulsiveness Scale, version 11 (Patton et al. 1995), *Y-BOCS* Yale-Brown Obsessive-Compulsive Scale (Goodman et al. 1989), *OCDUS* Obsessive-Compulsive Drug Use Scale (Franken et al. 2002)

^a^Fisher’s exact test

### General procedure

Participants attended three sessions, with 1 week between each session. The task was conducted an hour after a single dose of either placebo, a D_2/3_ agonist (pramipexole, 0.5 mg), or a D_2/3_ antagonist (amisulpride, 400 mg), timed to coincide with peak plasma concentrations. Three individuals with SUD received 1.5 mg of pramipexole. All subjects contributed data to the Bayesian analysis. One control participant contributed only placebo data and one participant with OCD contributed only amisulpride and pramipexole data, as they did not complete all three sessions. One subject from the SUD group, who contributed data from all three sessions, was excluded from Ersche et al. (2011) due to a behavioural performance cutoff. These three participants were not used for subsequent analyses correlating model parameters with symptoms, and with the key behavioural measures reported in Ersche et al. (2011). The experiment was conducted in an fMRI (functional magnetic resonance imaging) scanner, however the imaging data were not reanalysed here. Further details about the study procedure are described in Ersche et al. (2011).

### Serial probabilistic reversal learning task

**Fig. 1:**
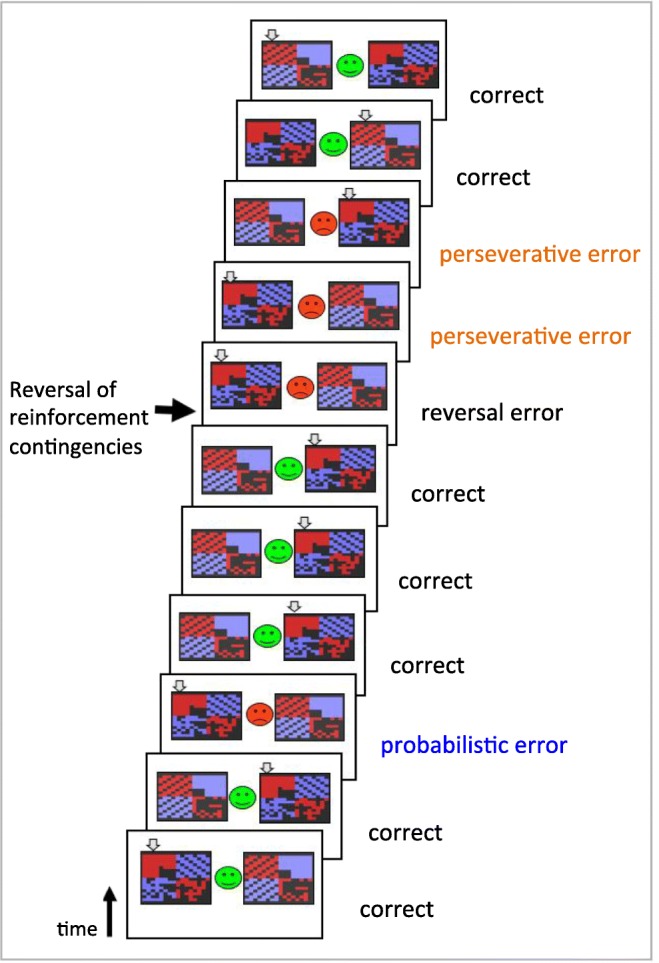
Schematic of the serial probabilistic reversal learning task, used with permission from Ersche et al. (2011). Two abstract stimuli were presented on either side of the screen, the participant selected one using a button press, and feedback was immediately given in the centre of the screen in the form of a green smiley face or a red frowning face. A probabilistic error occurred when a participant received spurious negative feedback after making the correct choice, which was rare and should therefore be ignored. A reversal error, on the other hand, was one where feedback was now truly negative, indicating the reversal had occurred, contingencies have thus changed and behaviour should be updated

Two visual stimuli were presented simultaneously, as shown in Fig. 1, and participants were prompted to make a choice by pressing one of two buttons. Stimuli were presented for 2000 milliseconds, and if a response was not entered in this period the screen would say “too late”. Participants received immediate feedback 500 ms after a response was made, in the form of a green face with a smile or a red face with a frown, and learned by trial and error which stimulus was correct most of the time. A fixation cross appeared between trials for a variable inter-trial interval lasting up to 3000 ms. Participants were told that intermittently they would receive negative feedback even if they made the correct choice, which they should ignore. Ignoring spurious minority feedback leads to more positive feedback overall, and is thus adaptive. They were also informed that the optimal response would reverse several times throughout the task: the initially correct response would lose its value and choosing the other stimulus would then be optimal. There were two runs of 10 sequences, making for 18 response reversals. Participants had to make at least 10 correct responses cumulatively before the contingencies reversed; this criterion varied from 10 to 15 to avoid participants anticipating the occurrence of a reversal. If, however, participants did not reach the required number of correct responses, the task stopped after the 200th trial of that run. Misleading negative feedback to a correct response was provided on about 15% of trials; this varied as a function of when the reversal occurred. Participants completed an initial practice run of 30 trials to familiarise themselves with the task.

Ersche et al. (2011) focused on three main behavioural measures in their conventional analysis: perseverative, probabilistic, and spontaneous errors. A perseverative error occurred when participants made at least one consecutive choice of the previously correct stimulus immediately after the reversal occurred, excluding any error on the first trial of the reversal. They calculated a perseverative error rate by dividing the number of perseverative errors by the number of sequences on which perseverative errors occurred. Probabilistic switches were inappropriate switches from the correct to incorrect stimulus following misleading negative feedback. Spontaneous errors occurred when participants switched from the correct to incorrect stimulus despite receiving veracious positive feedback. More probabilistic switches and spontaneous errors is analogous to more “lose-shift” and less “win-stay” behaviour, respectively—terms used in other studies (e.g. den Ouden et al. 2013; Rygula et al. 2015). Ersche et al. (2011) also reported the average number of trials per sequence.

### Computational modelling of behaviour

#### Overview

We fitted seven RL models to the behavioural data on PRL from Ersche et al. (2011) using hierarchical Bayesian methods, incorporating parameters that have been studied previously in the RL literature.

For all models, trials were sequenced across all trials in the PRL task. For each trial, the computational model was informed of the subject’s identity, the subject’s group and drug condition, which stimuli were presented and where (left or right side of the computer screen), the location (left or right) of the subject’s response, and whether the trial was rewarded or unrewarded.

**Fig. 2:**
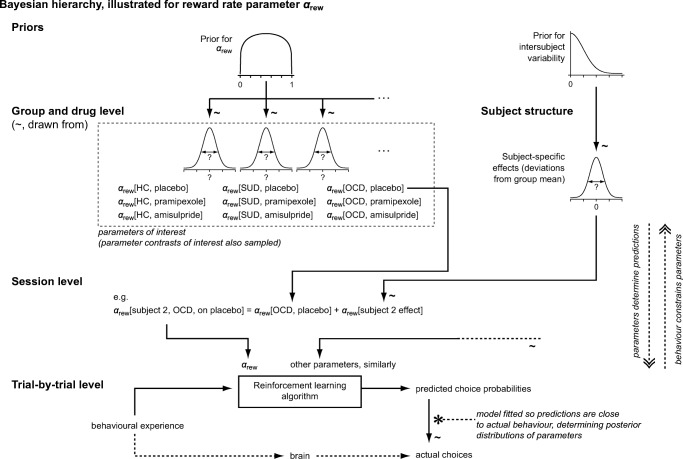
Schematic of the Bayesian hierarchy used in our analysis, illustrated here for a single parameter (reward rate). HC healthy controls

The top level of the Bayesian hierarchy (Fig. 2) pertained to group and drug: each RL parameter had a group- and drug-condition-specific distribution. The next level involved sessions for individual subjects: RL parameters for each subject in a given (drug) condition were drawn from a normal distribution whose mean was the group/drug mean (from the level above) and whose variance represents inter-subject variability for that parameter (implemented as a subject-specific deviation from the group/drug mean). Through this process, the computer established specific RL parameters for a given set of trials. It then used them to govern an RL model trained by the sequence of stimuli and reinforcement.

We define *t* as the trial number, *S*_*t*_ as the stimulus chosen on that trial, *L*_*t*_ as the location chosen on that trial, and *R*_*t*_ as the reinforcement delivered on that trial. Each stimulus was assigned an associated reinforcement-driven value *V*.

#### Models

Models are listed in Tables 2 and 3. Table 3 lists the models by order of complexity and nestedness.

**Table 2:** Model parameter prior distributions

	Models using each parameter	Prior	Reference, if applicable
Model parameters			
Reward learning rate, *α*^*rew*^	3, 4a, 4b, 4c	Beta(1.2, 1.2)	den Ouden et al. (2013)
Punishment learning rate, *α*^*pun*^	3, 4a, 4b, 4c	Beta(1.2, 1.2)	den Ouden et al. (2013)
Combined reward/punishment learning rate, *α*^*reinf*^	1, 2	Beta(1.2, 1.2)	den Ouden et al. (2013)
Reinforcement sensitivity, *τ*^*reinf*^	1, 2, 3, 4a, 4b, 4c, 5	Gamma(*α* = 4.82, *β* = 0.88)	Gershman (2016)
Location (side) stickness, *τ*^*loc*^	4a, 4c	Normal(0, 1)	Christakou et al. (2013)
Stimulus stickness, *τ*^*stim*^	2, 4b, 4c	Normal(0, 1)	Christakou et al. (2013)
Experience decay factor, *ρ*	5	Beta(1.2, 1.2)	den Ouden et al. (2013)
Decay factor for previous payoffs, *φ*	5	Beta(1.2, 1.2)	den Ouden et al. (2013)
Softmax inverse temperature, *β*	5 [note that *β =* 1 in all other models]	Gamma(*α* = 4.82, *β* = 0.88)	Gershman (2016)
Intersubject variability in parameters			
Intersubject standard deviations for *α*^*rew*^, *α*^*pun*^, *α*^*reinf*^*, τ*^*loc*^*, ρ, φ*	As above	Half-normal: Normal(0, 0.05) constrained to ≥0	
Intersubject standard deviations for *τ*^*reinf*^*, β*	As above	Half-normal: Normal(0, 1) constrained to ≥0	Gershman (2016) but altered from Cauchy to half-normal as per Stan recommendations (Stan Development Team; http://mc-stan.org/)

*rew* reward, *pun* punishment, *reinf.* reinforcement, *loc* location, *stim* stimulus

**Table 3:** Comparison of model performance

Rank	Name	Parameters	Log marginal likelihood	Log posterior P (model)
7	Model 1	*α*^*reinf*^, *τ*^*reinf*^	−16984.66	−503.8250
3	Model 2	*α*^*reinf*^, *τ*^*reinf*^, *τ*^*stim*^	−16687.72	−206.8821
6	Model 3	*α*^*rew*^, *α*^*pun*^, *τ*^*reinf*^	−16835.28	−354.4418
4	Model 4a	*α*^*rew*^, *α*^*pun*^, *τ*^*reinf*^, *τ*^*loc*^	−16732.50	−251.6656
2	Model 4b	*α*^*rew*^, *α*^*pun*^, *τ*^*reinf*^, *τ*^*stim*^	−16585.12	−104.2815
1	Model 4c	*α*^*rew*^, *α*^*pun*^, *τ*^*reinf*^, *τ*^*loc*^, *τ*^*stim*^	−16480.83	0.0000
5	Model 5	*ρ*, *φ*, *β*	−16821.35	−340.5171

Models are listed in order of increasing complexity and nestedness. Model ranked 1st was the winning model. Model names and parameters correspond to Table 2. The log marginal likelihood and log posterior P (model) are comparison metrics used to determine the best model. A numerically larger, i.e. less negative, log marginal likelihood is better. The prior probabilities of all models were equal

*rew* reward, *pun* punishment, *reinf.* reinforcement, *loc* location, *stim* stimulus

Model 1 employed two parameters and served to address whether a simple reinforcement learning algorithm was sufficient to best characterise behaviour between groups and under different drug conditions. Reinforcement led to an increase in value *V*_*i*_ of the stimulus *i* that was chosen, at a speed governed by the *reinforcement rate α*^*reinf*^, according to *V*_*i,t* + 1_ ← *V*_*i,t*_ + *α*^*reinf*^ (*R*_*t*_ – *V*_*i,t*_), where *R*_*t*_ represents the reward on trial *t* (by definition 1 on rewarded trials), and (*R*_*t*_ – *V*_*i,t*_) the prediction error. On nonrewarded trials *R*_*t*_ = 0, thus leading to a decrease in the value of *V*_*i*_. Stimulus value contributed to the final quantity controlling choice via *Q*^*reinf*^_*t*_ = *τ*^*reinf*^*V*_*t*_. The additional parameter *τ*^*reinf*^, termed *reinforcement sensitivity*, governs the degree to which a subject is driven by its reinforcement history. The quantities *Q* associated with the two available choices, for a given trial, were then fed into a standard softmax choice function to compute the probability of each choice:

$$ P\left({\mathrm{action}}_a\right)={\mathrm{softmax}}_{\beta}^a\left({Q}_1\dots {Q}_n\right)=\frac{e^{{\upbeta \mathrm{Q}}_a}}{\sum_{\mathrm{k}=1}^n{e}^{{\upbeta \mathrm{Q}}_k}} $$

for *n* = 2 choices with softmax inverse temperature *β* = 1. The probability values for each trial emerging from the softmax function (arbitrarily, the probability of choosing stimulus A) were fitted to the subject’s actual choices (did the subject choose stimulus A?). Note that since *β* = 1, the *τ* parameters directly represent weights given to each component in the softmax exponent.

Model 2 was as model 1 but additionally implementing the concept of “stimulus stickiness”, making for three parameters. This describes the tendency of a subject to respond again to a specific perceptual stimulus (regardless of location) that it chose on the previous trial, independent of outcome. This model served to test whether a combination of parameters that track both learning from action-outcome and stimulus-response associations could better describe behaviour. We added a *stimulus stickiness parameter τ*^*stim*^ and modelled this effect as *Q*^*stim*^_*t*_ = *τ*^*stim*^*s*_*t*–1_, where *s*_*t*–1_ was 1 for a stimulus that was chosen on the previous trial and 0 otherwise. The final quantity governing behaviour now incorporated this new component: *Q*_*t*_ = *Q*^*reinf*^_*t*_ + *Q*^*stim*^_*t*_. The quantities *Q*, associated with the two available choices for a given trial, were likewise fed into a standard softmax function as above.

Model 3 was as model 1, but instead of using one reinforcement rate *α*^*reinf*^, we implemented separate learning rates for rewarded outcomes, *α*^*rew*^, and nonrewarded outcomes, *α*^*pun*^. These separate parameters enabled us to test our prediction that groups would differ in how positive versus negative feedback guide behaviour, and to assess how dopaminergic agents modulated these processes. Reward led to an increase in value *V*_*i*_ of the stimulus *i* that was chosen, at a speed governed by the *reward rate α*^*rew*^, according to *V*_*i,t* + 1_ ← *V*_*i,t*_ + *α*^*rew*^ (*R*_*t*_ – *V*_*i,t*_), where *R*_*t*_ represents the reward on trial *t* (by definition 1 on rewarded trials), and (*R*_*t*_ – *V*_*i,t*_) the prediction error. Punishment (nonreward) led to a decrease in the value of *V*_*i*_ according to the *punishment rate α*^*pun*^, similarly: *V*_*i,t* + 1_ ← *V*_*i,t*_ + *α*^*pun*^ (*R*_*t*_ – *V*_*i,t*_) for *R*_*t*_ = 0. Stimulus value contributed to the final quantity controlling choice via *Q*^*reinf*^_*t*_ = *τ*^*reinf*^*V*_*t*_. Model 3 therefore had three parameters: *α*^*rew*^, *α*^*pun*^, and *τ*^*reinf*^, as per the “RP” model from den Ouden et al. (2013) (see Supplementary Material).

Model 4a was as model 3, while additionally implementing the concept of “side (location) stickiness”, a tendency to repeat responses to the side most recently chosen. This made for four parameters. We asked whether capturing a different perseverative tendency—behaviour bound to a location rather than a specific visual stimulus—in addition to learning from rewarded or unrewarded outcomes would better characterise behaviour. The tendency to choose a location was governed by the *location stickiness parameter τ*^*loc*^, according to *Q*^*loc*^_*l,t*_ = *τ*^*loc*^*L*_*l,t*–1_, where *L*_*l,t*–1_ represents the subject’s location choice on the previous trial (1 if *l* was the previously chosen location and 0 otherwise; for the first trial, this was 0 for both sides indicating no “stickiness”). The final tendency to choose a given stimulus at a given location was controlled by the quantity *Q*_*t*_ = *Q*^*reinf*^_*t*_ + *Q*^*loc*^_*t*_. This model thus led to quantities *Q* associated with the two available choices for a given trial.

Model 4b was the same as model 4a but implemented stimulus stickiness instead of side stickiness, giving four parameters. Given the SUD group from Ersche et al. (2011) perseverated to a particular stimulus, we predicted stimulus stickiness would be more informative than side stickiness.

Model 4c was as model 3 (*α*^*rew*^, *α*^*pun*^, and *τ*^*reinf*^) with the addition of parameters for both stimulus stickiness and side stickiness, giving five parameters. The final quantity governing behaviour was therefore *Q*_*t*_ = *Q*^*reinf*^_*t*_ + *Q*^*loc*^_*t*_ + *Q*^*stim*^_*t*_.

Model 5 used a different approach: experience-weighted attraction (EWA; Camerer and Ho 1999), which was the winning model in den Ouden et al. (2013) who used a single reversal. This model is described elsewhere (and in the Supplementary Material), but in brief, this model balances the value of incoming information against current beliefs (based on past experience). Learning from reinforcement is modulated by an “experience weight” for a stimulus; the experience weight for a stimulus is updated every time it is chosen, and its change over time is governed by a decay factor. In this model, the softmax inverse temperature *β* was also a parameter able to vary. The learning rate can decline over time in the EWA model. Because our paradigm employed serial reversals, requiring new learning at several points, it is possible this model may be more conducive to PRL with a single reversal.

### Priors, fitting, and model comparison

Priors for the parameters are shown in Table 2. Models were fitted using Hamiltonian Markov Chain Monte Carlo sampling via Stan 2.17.2 (Carpenter et al. 2017). Convergence was checked with the potential scale reduction factor measure $\hat{R}$ (Gelman et al. 2013; Brooks and Gelman 1998), which approaches 1 for perfect convergence; values below 1.2 are typically used as a guideline for convergence and a cutoff of < 1.1 is a stringent criterion for convergence (Brooks and Gelman 1998). The use of multiple simulation runs with measurement of convergence is an important check for simulation reliability (cf. Wilson and Collins 2019) and is an intrinsic part of Stan. We also verified parameter recovery from simulated data for the winning model (see Supplementary Material).

Models were compared using a bridge sampling estimate of the marginal likelihood (Gronau et al. 2017a) via the “bridgesampling” R package (Gronau et al. 2017b). This procedure directly estimates the marginal likelihood, and thus the posterior probability of each model, given the data, prior model probabilities, and the assumption that the models represent the entire family of those to be considered (Table 3). We assumed that all models had equal prior probability.

### Interpretation of results

In addition to the estimated parameters, comparisons of interest (e.g. between groups on placebo; between-group differences in the effects of each drug) were also sampled directly to give a posterior probability distribution for each quantity of interest. Posterior distributions were interpreted using the 95% highest posterior density interval (HDI), the Bayesian “credible interval”. Multiple comparisons correction procedures (which would be appropriate for a null-hypothesis significance testing approach) were not applied, since Bayesian hierarchical models intrinsically make comparisons more conservative through “shrinkage” of estimates drawn from a higher-level distribution (including, at the least, the priors at the top level), leading to an automatic multiple comparisons correction without the reduction in power seen with the classical approach (Gelman and Tuerlinckx 2000; Gelman et al. 2012; Kruschke 2011b).

We note in particular the following properties of the Bayesian modelling approach with respect to testing for group and drug effects. Firstly, identical priors were used for all group and drug conditions. Secondly, it is possible to examine group or drug differences via parameter estimation (e.g. calculating a posterior distribution on a group-difference parameter, and examining whether this plausibly includes zero) or model comparison (e.g. comparing a model that accounts for possible group differences with one that does not—compare Mkrtchian et al. (2017) for a related non-Bayesian approach from this perspective). Both approaches are superior to null hypothesis significant testing (Kruschke 2011b); however, the parameter estimation procedure is in general preferable to the model comparison approach (Kruschke 2011a, b). Allowing a model to measure a group difference does not bias the analysis to yield a nonzero group difference if there is none in the data (though, obviously, a model that does not represent “group” in its structure can never detect a group difference). Accordingly, we used the same model structure for group/drug comparisons throughout, and varied the model structure only for the RL aspects. Thirdly, a Bayesian analysis of a two-factor (e.g. group × drug) factorial design (Kruschke 2011b; Gelman et al. 2013) can be analysed in two equivalent ways: (a) with predictors corresponding to the traditional coding of an ANOVA design matrix [intercept, group effect(s), drug effect(s), interaction effect(s)], whose coefficient-weighted combination determines the expected value for a cell, or (b) a “cell means” approach with the mean for each factor combination (each cell) estimated directly (e.g. control/placebo mean, control/amisulpride mean, OCD/placebo mean, etc.). These methods are equivalent (and have identical numbers of parameters). We chose the latter as this makes obtaining posterior distributions for drug/group effects simpler; it also improves the computational implementation, as parameter range constraints can be directly specified and the Monte Carlo process can be allowed to explore distributions that are less interdependent. Fourthly, in all cases it is critical that the model design correctly captures the correlation structure of the data, such as the within-subjects structure.

### Simulation of behavioural data from winning model

To establish if the winning model was sufficient to reproduce key behavioural phenomena, we simulated behavioural data from the winning model, and analysed it as per Ersche et al. (2011). For each group (healthy controls, SUD, OCD) and drug (placebo, amisulpride, pramipexole) combination, we simulated 100 identical virtual “subjects” using the posterior group mean parameters from the winning model. Each “subject” performed the probabilistic reversal learning task in silico. We did not simulate inter-subject parameter variability (or, therefore, a within-subjects structure), because the purpose of this analysis is to use arbitrarily high power to establish the model’s sufficiency to reproduce known behavioural patterns. For a given group/drug combination, variability in the decisions made by each virtual subject is a consequence only of the random process via which choice probabilities are mapped to concrete choices, and the random assignment of stimuli to left/right sides. Source code is presented in the Supplementary Material, as is a full description of the simulated task.

To demonstrate the necessity (as well as the sufficiency) of changes in stickiness parameters to explain key behavioural effects, we conducted two further simulations. The first additional simulation fixed the location stickiness parameter, *τ*^*loc*^, so that it did not vary between groups or drugs. The simulation was performed exactly as above except that for all “subjects” in all drug conditions, *τ*^*loc*^ was set to its overall posterior mean (taken, for simplicity, as the mean of the 3 × 3 per-group/per-drug posterior mean parameters). The second additional simulation did the same but fixed *τ*^*stim*^ instead, likewise.

## Results

### Baseline characteristics

Whilst participants did not have any other Axis I psychiatric diagnosis at the time of the study, the groups differed in their depression scores on the Beck Depression Inventory (BDI-II; Beck et al. 1996; *F*_(2,50)_ = 19.782, *p* < 0.001). Both the SUD and OCD groups had significantly greater depression scores than controls (*t*(18) = − 3.759, *p* = 0.001 for SUD; t(18) = − 5.960, *p* < 0.001 for OCD). Depression scores in the OCD group were also significantly greater than in the SUD group (*t*(28) = − 3.068, *p* = 0.005). Other baseline characteristics on the three groups of participants are presented in Table 1.

### Choice of model

**Fig. 3:**
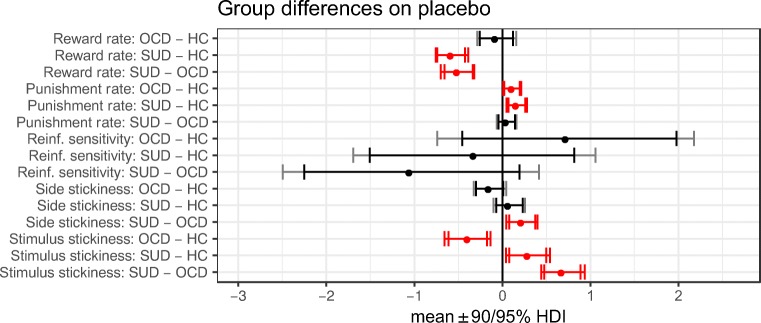
Differences between groups on placebo. Reinf. reinforcement, HC healthy controls. The optimal computational model contained parameters measuring (from top to bottom) learning from positive feedback, learning from negative feedback, sensitivity to reinforcement, a tendency to repeat choices on a recently chosen side (side stickiness), and a tendency to repeat choices to a recently chosen stimulus (stimulus stickiness). Differences in parameter per-group means under placebo; posterior 0 ∉ 95% HDI signified in red

**Fig. 4:**
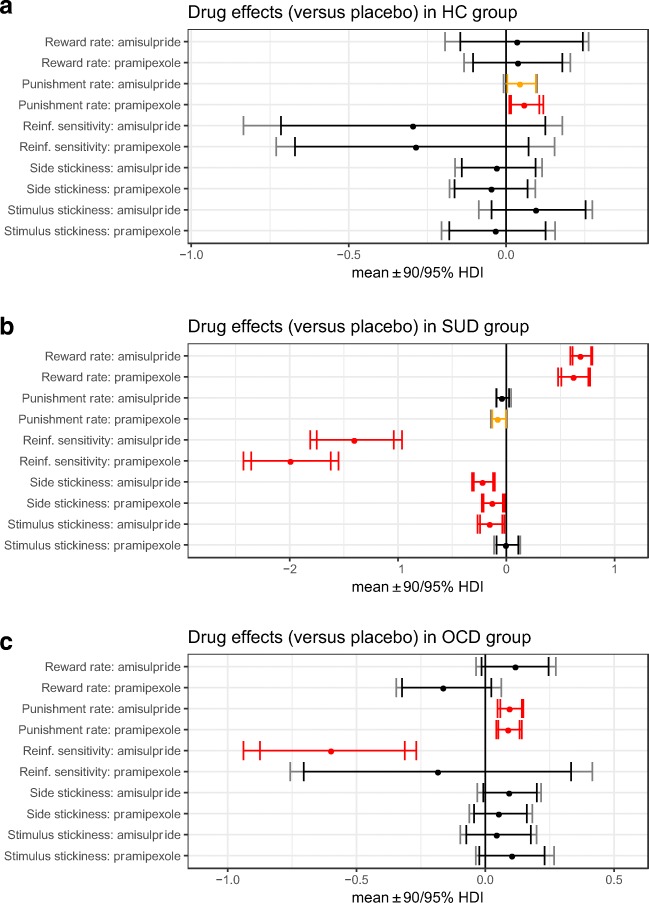
Effects of amisulpride and pramipexole (relative to placebo) in each group. Reinf. reinforcement, HC healthy controls. **a** Difference in parameter per-condition means between HC on amisulpride or pramipexole compared to HC on placebo; posterior 0 ∉ 95% HDI signified in red, 0 ∉ 90% HDI in orange; HC_drug – HC_placebo. **b** Difference in parameter per-condition means between SUD under amisulpride or pramipexole compared to SUD on placebo, posterior 0 ∉ 95% HDI; SUD_drug – SUD_placebo. **c** Difference in parameter per-condition means between OCD on amisulpride or pramipexole compared to OCD on placebo, posterior 0 ∉ 95% HDI; OCD_drug – OCD_placebo

**Fig. 5:**
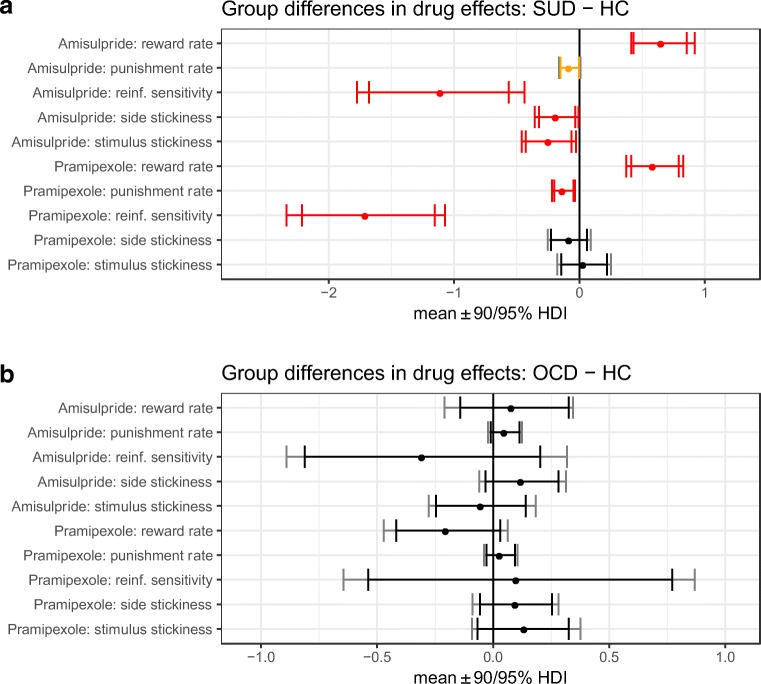
Differences in the effects of amisulpride or pramipexole between patient groups and healthy controls. All contrasts represent the difference between drug X’s effect in the patient group and its effect in the control group. Reinf. reinforcement, HC healthy controls. **a** [(SUD_drug – SUD_placebo) – (HC_drug – HC_placebo)] for amisulpride, and separately for pramipexole; posterior 0 ∉ 95% HDI signified in red, 0 ∉ 90% HDI in orange. **b)** Posterior 0 ∈ 95% HDI denoted in black, indicating no differences. This was a subtraction of [(OCD_drug – OCD_placebo) – (HC_drug – HC_placebo)] for amisulpride, and separately for pramipexole

A simple computational model of reinforcement learning best described behaviour. Conventional analyses of PRL assess sensitivity to immediate reinforcement (Murphy et al. 2003; Ersche et al. 2011) and do not account for the possibility that choice behaviour is influenced by an integration of feedback history from multiple experiences (Rygula et al. 2015). To assess the computations underlying task performance, beyond the influence of immediate feedback, we fitted and compared seven reinforcement learning models (Table 2). Convergence was not perfect, with a maximum $\hat{R}$ = 1.478, but was good, with > 99% of parameters and contrasts having $\hat{R}$ < 1.1**.** Moreover, all parameters of interest (all group-level mean and distributional parameters and all contrasts) had $\hat{R}$ < 1.121. The winning model, as determined using a bridge sampling estimate of the marginal likelihood (Table 3), included five parameters: (1) positive reinforcement rate, the extent to which behaviour is driven by learning from positive feedback; (2) negative reinforcement rate, or learning from negative feedback; (3) reinforcement sensitivity, which is the overall sensitivity to reinforced stimulus value; (4) “stimulus stickiness”, the tendency to repeat choices to recently chosen stimuli, regardless of outcome; and (5) “side (location) stickiness”, the degree to which participants responded to, or got “stuck” to, the same side (location) of the computer screen as before, left or right, irrespective of stimulus or outcome. The stickiness parameters, it is worth noting, can be comparable to strategies of exploration versus exploitation (Clarke et al. 2014; Seymour et al. 2012) and may relate to conventional measures of perseveration. The winning model demonstrated parameter recovery from simulated data (see Supplementary Material). All results are summarised in Tables 3, 4 and 5 and in Figs. 3, 4 and 5.

**Table 4:** Between-group effects on parameters from the winning model

	SUD vs HC			OCD vs HC			SUD vs OCD
Parameter	Placebo	Effects of amisulpride	Effects of pramipexole	Placebo	Effects of amisulpride	Effects of pramipexole	Placebo
Reward learning rate, *α*^*rew*^	↓	↑	↑				↓
Punishment learning rate, *α*^*pun*^	↑		↓	↑			
Reinforcement sensitivity, *τ*^*reinf*^		↓	↓				
Location (side) stickness, *τ*^*loc*^		↓	↓				↑
Stimulus stickness, *τ*^*stim*^	↑	↓		↓			↑

HC healthy controls. Contrasts shown are (left to right) SUD_placebo – HC_placebo; [(SUD_drug – SUD_placebo) – (HC_drug – HC_placebo)] for amisulpride, and separately for pramipexole; OCD_placebo – HC_placebo; [(OCD_drug – OCD_placebo) – (HC_drug – HC_placebo)] for amisulpride, and separately for pramipexole; SUD_placebo – OCD_placebo. These results correspond to Figs. 3 and 5a, b. Arrows denote an increase or decrease of a parameter in a given contrast. Lack of an arrow indicates no difference

**Table 5:** Within-group drug effects on parameters from the winning model

	HC		SUD		OCD	
Parameter	Amisulpride	Pramipexole	Amisulpride	Pramipexole	Amisulpride	Pramipexole
Reward learning rate, *α*^*rew*^			↑	↑		
Punishment learning rate, *α*^*pun*^	↑	↑		↓	↑	↑
Reinforcement sensitivity, *τ*^*reinf*^			↓	↓	↓	
Location (side) stickness, *τ*^*loc*^			↓	↓		
Stimulus stickness, *τ*^*stim*^			↓			

All effects are comparisons between drug and placebo within a group. HC healthy controls. Within group comparisons: HC_amisulpride – HC_ placebo; HC_pramipexole – HC_placebo; likewise for SUD and OCD. These results correspond to Fig. 4a–c. Arrows denote an increase or decrease of a parameter in a given contrast. Lack of an arrow indicates no difference

### Simulation of behavioural data from winning model

The winning model reproduced key behavioural phenomena found by Ersche et al. (2011). In line with their results, our analysis of simulated data revealed a main effect of group for the number of trials per sequence (*F*_(2,297)_ = 97.477, *p* = 2.84 × 10^−33^). The SUD group required more trials per sequence to reach criterion compared to both the healthy control group (*t*_(195)_ = − 8.792, *p* = 7.62 × 10^−16^) and the OCD group (*t*_(187)_ = − 13.638, *p* = 7.36 × 10^−30^). The groups also differed in the number of spontaneous errors (*F*_(2,297)_ = 394.392, *p* = 2.49 × 10^−84^), with the SUD group making more spontaneous errors than both controls (*t*_(168)_ = − 21.028, *p* = 5.80 × 10^−49^) and the OCD group (*t*_(148)_ = − 23.446, *p* = 1.0504 × 10^−51^).

There was a main effect of group, absent in Ersche et al. (2011), for the number of probabilistic switches (*F*_(2,297)_ = 26.896, *p* = 1.84 × 10^−11^). Both the SUD (*t*_(198)_ = − 4.302, *p* = 2.7 × 10^−5^) and OCD groups (*t*_(198)_ = − 7.343, *p* = 5.31 × 10^−12^) showed greater probabilistic switching compared to controls, and the OCD group demonstrated more probabilistic switching than the SUD group (*t*_(198)_ = − 3.032, *p* = 0.003). We observed a main effect of perseverative error rate (*F*_(2,297)_ = 36.101, *p* = 9.24 × 10^−15^). While this main effect was absent in Ersche et al. (2011), our follow up *t* tests are in line with their post hoc analysis: The perseverative error rate was greater in SUD compared to controls (*t*_(183)_ = − 2.385, *p* = 0.018), was greater in SUD compared to OCD (*t*_(173)_ = 7.967, *p* = 2.10 × 10^−13^), and was greater in healthy controls than in OCD (*t*_(198)_ = 6.611, *p* = 3.46 × 10^−10^).

Next, we assessed drug effects in our simulated data, again using the same analyses as reported in Ersche et al. (2011). There was a significant effect of drug on number of trials per sequence (*F*_(2,891)_ = 6.603, *p* = 0.001), and there was also a drug-by-group interaction (*F*_(4,891)_ = 6.234, *p* = 6 × 10^−5^). For spontaneous errors, there was also a main effect of drug (*F*_(2,891)_ = 33.939, *p* = 6.24 × 10^−15^) and a drug-by-group interaction (*F*_(4,891)_ = 22.624, *p* = 8.09 × 10^−18^). There was a main effect of drug on probabilistic switches as well (*F*_(2,891)_ = 10.802, *p* = 2.3 × 10^−5^). As in Ersche et al. (2011), there was no drug-by-group interaction (*F*_(4,891)_ = 1.630, *p* = 0.165) for probabilistic switches. We found a drug-by-group interaction on the perseverative error rate (*F*_(4,891)_ = 3.377, *p* = 0.009), in line with Ersche et al. (2011). Post hoc analyses on the perseverative error rate revealed a main effect of group for amisulpride (*F*_(2,297)_ = 14.939, *p* = 6.58 × 10^−7^) and for pramipexole (*F*_(2,297)_ = 20.347, *p* = 5.23 × 10^−9^). In contrast to Ersche et al. (2011), pramipexole was not associated with a change in the perseverative error rate compared to placebo, in SUD (*t*_(198)_ = − 0.071, *p* = 0.944). The difference in the perseverative error rate in the SUD group and healthy controls persisted when on pramipexole (*t*_(178)_ = − 3.933, *p* = 1.20 × 10^−4^). Consistent with Ersche et al. (2011), there was no change in the perseverative error rate on pramipexole relative to placebo in healthy controls (*t*_(198)_ = 1.912, *p* = 0.057) or in the OCD group (*t*_(198)_ = − 0.980, *p* = 0.328). Amisulpride, in line with Ersche et al. (2011), did not significantly alter the perseverative error rate compared to placebo in the healthy control (*t*_(198)_ = − 1.147, *p* = 0.253), SUD (*t*_(198)_ = 1.618, *p* = 0.107), or OCD (*t*_(190)_ = − 1.876, *p* = 0.062) groups.

### Simulation with fixed value for stimulus stickiness

Next, we analysed simulated data generated with a fixed value for the stimulus stickiness parameter, to determine the whether variation in this parameter was necessary to optimally capture the key behavioural phenomena from Ersche et al. (2011). Whilst on placebo, the main effects of group persisted for the number of trials per sequence (*F*_(2,297)_ = 88.209, *p* = 8.52 × 10^−31^) and spontaneous errors (F_(2,297)_ = 911.582, *p* = 1.73 × 10^−127^). The SUD group still required a greater number of trials per sequence than the healthy controls (*t*_(198)_ = − 10.195, *p* = 6.91 × 10^−20^) and the OCD group (*t*_(198)_ = − 12.182, *p* = 7.66 × 10^−26^), and this was also true for spontaneous errors: there were more spontaneous errors in SUD compared to controls (*t*_(178)_ = − 31.738, *p* = 3.09 × 10^−75^) and relative to the OCD group (*t*_(157)_ = − 36.893, *p* = 2.34 × 10^−79^). There was a main effect of group on probabilistic switches (*F*_(2,297)_ = 39.424, *p* = 6.53 × 10^−16^), which was absent in Ersche et al. (2011), but present in the prior simulation analysis which incorporated variation in stimulus stickiness parameter. The pattern of effects, however, changed. In the standard simulation analysis, above, the OCD group showed the most probabilistic switches, followed by the SUD group. When fixing the stimulus stickiness parameter value, however, the SUD group now displayed the most probabilistic switching. There were more probabilistic switches in the SUD group compared to controls (*t*_(198)_ = − 8.336, *p* = 1.28 × 10^−14^) and in SUD compared to the OCD group (*t*_(198)_ = − 6.654, *p* = 2.73 × 10^−10^). Probabilistic switches in the OCD group, at the same time, were no longer significantly greater than controls (*t*_(198)_ = − 1.705, *p* = 0.09), which had been the case when incorporating variation in the stimulus stickiness parameter. Critically, the main effect of group on the perseverative error rate, in simulated data generated without variation in the stimulus stickiness parameter, was completely abolished (*F*_(2,297)_ = 0.546, *p* = 0.58). This finding on the perseverative error rate underlines the importance and centrality of the stimulus stickiness parameter in our model.

Critically, by fixing the value of the stimulus stickiness parameter, there was no main effect of drug (*F*_(2,897)_ = 0.004, *p* = 0.996), nor was there a drug-by-group interaction (*F*_(4,891)_ = 2.322, *p* = 0.055) on the perseverative error rate. The main findings of Ersche et al. (2011) hinged on the presence of this interaction. We therefore underline the importance of variation in the stimulus stickiness parameter in our model, by showing it is necessary to reproduce key patterns of effects on behaviour in relation to drug and disease, seen in the original data.

### Simulation with fixed value for side stickiness

We then analysed simulated data generated instead with a fixed value for the side (location) stickiness parameter, with variation in the stimulus stickiness parameter. In contrast to the importance of variation in the stimulus stickiness parameter to capture an effect of group on the perseverative error rate, a fixed value of the side stickiness parameter still produced a highly significant effect of group on the perseverative error rate under placebo (*F*_(2,297)_ = 28.519, *p* = 4.68 × 10^−12^). The SUD group had a higher perseverative error rate than both the healthy controls (*t*_(171)_ = − 4.145, *p* = 5.3 × 10^−5^) and the OCD group (*t*_(198)_ = − 6.977, *p* = 4.43 × 10^−11^). Controls additionally had a greater perseverative error rate than the OCD group (*t*_(198)_ = 3.692, *p* = 2.87 × 10^−4^). The three other key behavioural patterns could also be captured despite a fixed value for the side (location) stickiness parameter: number of trials per sequence (*F*_(2,297)_ = 147.069, *p* = 4.06 × 10^−45^) and spontaneous errors (*F*_(2,297)_ = 484.526, *p* = 3.09 × 10^−94^)—both consistent with Ersche et al. (2011)—as well as probabilistic switches (*F*_(2,297)_ = 27.327, *p* = 1.28 × 10^−11^). In line with Ersche et al. (2011), the SUD group required more trials per sequence than both the healthy controls (*t*_(191)_ = − 12.046, *p* = 3.19 × 10^−25^), and the OCD group (*t*_(187)_ = − 15.925, *p* = 1.26 × 10^−36^), and the same pattern was observed for spontaneous errors: the SUD group made more spontaneous errors than the healthy controls (*t*_(170)_ = − 24.657, *p* = 4.30 × 10^−58^) and the OCD group (*t*_(169)_ = − 25.448, *p* = 1.02 × 10^−59^). Pairwise comparisons on the number of probabilistic switches produced the same pattern of results as in our first simulation analysis, where neither stimulus nor side stickiness were fixed values: Both the SUD (*t*_(198)_ = − 4.861, *p* = 2 × 10^−6^) and OCD groups (*t*_(183)_ = − 7.484, *p* = 2.93 × 10^−12^) showed greater probabilistic switching compared to controls, and the OCD group demonstrated more probabilistic switching than the SUD group (*t*_(198)_ = 2.547, *p* = 0.012).

Critically, by holding constant the value of the side (location) stickiness parameter, there was no main effect of drug (*F*_(2,897)_ = 0.937, *p* = 0.392), nor was there a drug-by-group interaction on the perseverative error rate (*F*_(4,891)_ = 2.047, *p* = 0.086), demonstrating the importance of variation in this model parameter.

### Stimulus stickiness

Our first novel result was that the stimulus stickiness parameter differentiated SUD and OCD. Stimulus stickiness assesses stimulus-bound behaviour, a measure of the degree to which choices were driven by the stimulus that was selected in the recent past, irrespective of outcome. Under placebo, individuals with SUD showed significantly increased stimulus stickiness relative to healthy controls (difference in parameter per-group mean, posterior 95% highest posterior density interval (HDI) excluding zero), whereas people with OCD showed decreased stimulus stickiness relative to controls (group difference, 0 ∉ 95% HDI); see Table 4 and Fig. 3.

### Effects of dopaminergic agents on stimulus stickiness

Amisulpride ameliorated the elevated stimulus stickiness in SUD: there was improvement both compared to their performance on placebo (drug difference, 0 ∉ 95% HDI; Fig. 4b; Table 5), and relative to the effects of amisulpride on healthy controls (group difference in drug effect, 0 ∉ 95% HDI; Fig. 5a; Table 4). Stimulus stickiness in OCD, on the other hand, was unaffected: amisulpride did not have a different effect on stimulus stickiness in OCD relative to their performance on placebo (no drug difference, 0 ∈ 95% HDI; Fig. 4c; Table 5) nor when compared to the effect of amisulpride on healthy controls (no group difference, 0 ∈ 95% HDI; Fig. 5b; Table 4). Pramipexole had no effect on stimulus stickiness in SUD or OCD (no differences 0 ∈ 95% HDI; Figs. 4b, c and 5a, b; Tables 4 and 5). Stimulus stickiness in healthy controls was unaffected by either drug (no drug differences, 0 ∈ 95% HDI; Fig. 4a; Table 5).

### Side (location) stickiness

We also evaluated another type of stickiness: side stickiness, or the tendency to repeat choices to the same side (location) of the computer screen as before, regardless of stimulus type or outcome. Side (location) stickiness was greater in SUD compared to OCD (group difference, 0 ∉ 95% HDI; Fig. 3; Table 4), and dopaminergic agents modulated this parameter in SUD only. Individuals with OCD, on placebo, showed no impairment of side stickiness relative to healthy controls (no group difference, 0 ∈ 95% HDI; Fig. 3; Table 4). Both amisulpride and pramipexole reduced side stickiness in individuals with SUD compared to their performance on placebo (drug differences, 0 ∉ 95% HDI; Fig. 4b; Table 5). When comparing drug effects in SUD to those in controls, amisulpride reduced side stickiness more in SUD than in controls (group difference, 0 ∉ 95% HDI; Fig. 5a; Table 4), but pramipexole did not have a differential effect (no group difference, 0 ∈ 95% HDI; Fig. 5a; Table 4). In the OCD group, side stickiness was unaffected by either amisulpride or pramipexole compared to placebo (no drug differences, 0 ∈ 95% HDI; Fig. 4c; Table 5). Amisulpride and pramipexole, additionally, did not differentially affect side stickiness in the OCD group relative to healthy controls (no group differences, 0 ∈ 95% HDI; Fig. 5b; Table 4).

### Reward-driven learning

We measured the rate at which participants learned from positive feedback in the task. Reward-driven learning was impaired in SUD and not in OCD: on placebo, individuals with SUD showed diminished learning from positive feedback relative to controls (group difference, 0 ∉ 95% HDI; Fig. 3; Table 4), while the OCD group was no different from healthy controls in their reward learning (no group difference, 0 ∈ 95% HDI; Fig. 3; Table 4). Reward learning in SUD was sensitive to dopaminergic agents: Both amisulpride and pramipexole remediated the diminished reward learning seen under placebo, increasing reward-driven learning (drug differences, 0 ∉ 95% HDI; Fig. 4b; Table 5). This was also the case when comparing the effects of the dopaminergic agents on reward-driven learning in SUD versus healthy controls (group differences, 0 ∉ 95% HDI; Fig. 5a; Table 4). Neither amisulpride nor pramipexole, meanwhile, altered reward-driven learning in individuals with OCD, both when compared to these drugs’ effects in controls (no group differences, 0 ∈ 95% HDI; Fig. 5b; Table 4) and when contrasted with their own performance on placebo (no drug differences, 0 ∈ 95% HDI; Fig. 4c; Table 5). Reward learning in controls was unaffected by amisulpride and pramipexole, compared to placebo (no drug differences, 0 ∈ 95% HDI; Fig. 4a; Table 5).

### Punishment-driven learning

Both individuals with SUD and with OCD showed increased learning from negative feedback (punishment in the form of nonreward) on placebo, compared to healthy controls (group differences, 0 ∉ 95% HDI; Fig. 3; Table 4). In individuals with SUD, pramipexole led to a small improvement in their elevated negative feedback learning relative to placebo (drug difference, 0 ∉ 90% HDI; Fig. 4b; Table 5). Amisulpride, on the other hand, neither worsened nor corrected the elevated negative feedback-driven learning seen on placebo (no drug difference, 0 ∈ 95% HDI; Fig. 4b; Table 5). Relative to their baseline performance on placebo, the OCD group showed a further increase in learning from negative feedback on both amisulpride and pramipexole (drug differences, 0 ∉ 95% HDI; Fig. 4c; Table 5). The negative feedback learning rate was the only parameter in the model that was modulated in healthy controls by dopaminergic agents. The control group showed an increase in learning from negative feedback on amisulpride (drug difference, 0 ∉ 90% HDI; Fig. 4a; Table 5) and more so under pramipexole (drug difference, 0 ∉ 95% HDI; Fig. 4a; Table 5). Amisulpride and pramipexole, in fact, increased negative feedback-driven learning in OCD and healthy controls to the same extent (no group differences, 0 ∈ 95% HDI; Fig. 5b; Table 4). Dopaminergic agents differentially affected negative feedback learning in SUD when contrasted with healthy controls: the SUD group showed a relative decrease in this parameter on amisulpride (group difference, 0 ∉ 90% HDI; Fig. 5a; Table 4) and pramipexole (group difference, 0 ∉ 95% HDI; Fig. 5a; Table 4), driven in part by the drug-induced elevated negative feedback learning rate in controls.

### Reinforcement sensitivity

Reinforcement sensitivity, the overall sensitivity to reinforced stimulus value, was unimpaired in SUD and OCD at baseline but was compromised by dopaminergic agents. Results on this parameter were not different between the OCD, SUD, or healthy control groups under placebo (no group differences, 0 ∈ 95% HDI; Fig. 3; Table 4). There were, however, drug-induced effects. Reinforcement sensitivity in individuals with SUD was most impaired by dopaminergic modulation. Both amisulpride and pramipexole reduced reinforcement sensitivity in SUD, relative to placebo (group differences, 0 ∉ 95% HDI; Fig. 4b; Table 5). Amisulpride and pramipexole also reduced reinforcement sensitivity in SUD when compared to the drug effects on healthy controls (drug differences, 0 ∉ 95% HDI; Fig. 5a; Table 4). In the OCD group, amisulpride induced a deficit compared to placebo (drug difference, 0 ∉ 95% HDI; Fig. 4c; Table 5), whereas pramipexole had no effect (no drug difference, 0 ∈ 95% HDI; Fig. 4c; Table 5). Amisulpride and pramipexole did not differentially affect reinforcement sensitivity in the OCD group compared to controls (no group differences, 0 ∈ 95% HDI; Fig. 5b; Table 4). Finally, neither amisulpride nor pramipexole induced an impairment of reinforcement sensitivity in healthy controls compared to placebo (no drug differences, 0 ∈ 95% HDI; Fig. 4a, Table 5).

### Correlations with conventional behavioural measures

We next tested to see how the parameters in our winning model related to the conventional measures of behaviour (see “Methods”) from Ersche et al. (2011). Because stimulus stickiness is a measure of stimulus-bound perseveration, we asked whether stimulus stickiness was correlated with either conventional measure of perseveration in Ersche et al. (2011), which was their primary focus. We found that in healthy controls on placebo, stimulus stickiness was significantly positively correlated with the number of perseverative errors (*r* = 0.587, *p* = 0.01, uncorrected) and the perseverative error rate (*r* = 0.672, *p* = 0.002, uncorrected). In SUD and OCD on placebo, however, stimulus stickiness was not correlated with the perseverative error rate or the number of perseverative errors (all *p* > 0.05). Under amisulpride or pramipexole, moreover, neither the perseverative error rate nor the number of perseverative errors correlated with stimulus stickiness in the healthy control, SUD, or OCD groups (all *p* > 0.05).

Side stickiness was not correlated with either measure of perseveration under placebo in the healthy control, SUD, or OCD groups (all *p* > 0.05). There were also no significant correlations between side stickiness and the two measures of perseveration in the SUD or OCD groups on either amisulpride or pramipexole (all *p* > 0.05). In healthy controls on amisulpride there was a significant correlation between side stickiness and the number of perseverative errors (*r* = 0.773, *p* = 1.69 × 10^−4^, uncorrected), and the perseverative error rate (*r* = 0.473, *p* = 0.048, uncorrected). On pramipexole, side stickiness was also correlated in healthy controls with the number of perseverative errors (*r* = 0.499, *p* = 0.035, uncorrected).

We also asked whether the diminished reward learning rate we observed in SUD on placebo was related to the increase in spontaneous errors reported in Ersche et al. (2011)—in other words a decrease in staying with the correct choice despite having received a reward (decreased win-stay). Indeed, a decreased reward rate was correlated with a greater number of spontaneous errors in SUD on placebo (*r* = − 0.752, *p* = 4.92 × 10^−4^, uncorrected). We then asked whether an increased punishment learning rate was related to increased probabilistic switches in Ersche et al. (2011)—increased switching to the incorrect choice following misleading negative feedback, i.e. lose-shift behaviour. This was indeed the case for all three groups: a greater punishment learning rate was correlated with more probabilistic switches in healthy controls (*r* = 0.748, *p* = 3.58 × 10^−4^, uncorrected), SUD (*r* = 0.815, *p* = 6.7 × 10^−5^, uncorrected), and OCD (*r* = 0.858, *p* = 5 × 10^−6^, uncorrected). Further correlations between the model parameters and conventional behavioural measures are presented in the Supplementary Material.

### Pairwise tests of probabilistic switches

Because Ersche et al. (2011) did not find a main effect of group on probabilistic switching (lose-shift) behaviour they did not report pairwise comparisons between the groups. We realised conducting these comparisons would be important for multiple reasons: It would enable us to (1) compare the original SUD data from Ersche et al. (2011) to PRL in alcohol use disorder (AUD) from Reiter et al. (2016) who compared only two groups; (2) compare the original SUD data to the results from our simulation analysis; (3) compare the original OCD data to that of Hauser et al. (2017), who also only compared two groups; and (4) to understand how the original findings in the healthy control, SUD, and OCD groups relate to parameters such as the punishment learning rate (see Table 4 in the Supplementary Materials). The SUD group showed more probabilistic switching (lose-shift) behaviour compared to healthy controls (*t*_(33)_ = − 2.119, *p* = 0.042). This effect was also marginally significant in the OCD group compared to controls: increased probabilistic switching (*t*_(23)_ = − 2.054, *p* = 0.051). There was no difference between the OCD and SUD groups (*t*_(27)_ = − 0.606, *p* = 0.549).

### Correlations with questionnaire measures

We tested whether stimulus-bound behaviour—stimulus stickiness—as measured under placebo, was correlated with severity of compulsive symptoms as assessed through questionnaires. Scores on the Obsessive Compulsive Drug Use Scale (OCDUS; Franken et al. 2002) in the SUD group were not correlated with their stimulus stickiness results (*r* = − 0.028, *p* = 0.914). There was also no significant correlation between stimulus stickiness in the OCD group and their scores on the Yale-Brown Obsessive Compulsive Scale (Y-BOCS, Goodman et al. 1989; *r* = − 0.395, *p* = 0.105). We also tested whether stimulus stickiness in SUD was correlated with years of drug use, which would suggest a drug-induced effect; this correlation, however, was not significant (*r* = − 0.177, *p* = 496). Because the SUD, OCD, and control groups differed in their depression scores on the BDI-II, we also tested whether this was correlated with stimulus stickiness results on placebo. Depression scores were not correlated with stimulus stickiness in SUD (*r* = − 0.163, *p* = 0.531), OCD (*r* = 0.415, *p* = 0.087), or healthy controls (*r* = − 0.080, *p* = 0.754).

## Discussion

The aim of our study was to uncover the microstructure of behaviour, and its dopaminergic modulation, in SUD and OCD using RL models. We found that the computational profile of PRL performance differed between SUD, OCD, and healthy controls, and both dopaminergic drugs tested modulated behavioural parameters in all three groups (Figs. 3, 4 and 5; Tables 4 and 5), which considerably extends the conventional analyses of Ersche et al. (2011). One key result was in regard to stimulus stickiness, which measures a basic perseverative tendency. This one measure differentiated all three groups: individuals with SUD demonstrated increased stimulus stickiness while the OCD group displayed a decrease relative to controls (Fig. 3). The former result is consistent with Ersche et al. (2011) who showed a perseverative impairment in SUD. The finding of the opposite change in OCD, meanwhile, demonstrates that the stimulus stickiness measure in our model was able to detect subtleties of behaviour across diagnostic categories which were not clearly delineated using conventional methods. Interestingly, stimulus stickiness only correlated with perseveration in our healthy control group, suggesting that this measure is indeed related to perseveration, yet also reinforcing the novelty of stimulus stickiness in assessing these two disorders of compulsivity. This is in consonance with Rygula et al. (2015) who studied PRL in monkeys with different serotonergic lesions: compared to controls they found elevation of stimulus stickiness in one group, reduction in another, and perseveration in neither. A second major set of findings related to basic changes in reward and punishment (nonreward) learning occurring in the SUD and OCD groups, not formalised in the earlier study. The SUD group showed reduced reward learning, whilst both groups demonstrated enhanced learning rates with punishment. These parameters were also sensitive to dopaminergic drug treatments and are discussed in detail below.

### Computational modelling procedure

We used a fully Bayesian process for model comparison and parameter estimation. The theoretically optimal method for model comparison is to evaluate the probabilities of each model, given the data; such a process incorporates Occam’s razor correctly, penalizing over-complex models (Kruschke 2011a, b; MacKay 2003; Gronau et al. 2017a). Bridge sampling (Gronau et al. 2017a, b) allows these probabilities to be estimated directly, in combination with prior probabilities of models; we assumed all models were equiprobable a priori. In our study, this process eliminated simple reinforcement learning algorithms and selected a model incorporating reinforcement learning with separate rates for reward and punishment (nonreward), in addition to perseverative (“stickiness”) behaviour in respect both of the stimulus selected and the response side (location). This model was superior to the EWA model, which incorporates perseverative tendencies in a different way. The model comparison process only selects amongst the models offered for comparison; it is of course inevitable that the true biological processes are more complex than our winning model, and possible that a more complex model that we did not consider was better. Nevertheless, simulations demonstrated that the winning model was sufficient (and variation in its stickiness parameters necessary) to capture the key behavioural phenomena found in this dataset by Ersche et al. (2011). Critically, group differences in perseveration were completely abolished in simulated data generated without variation in the stimulus stickiness parameter. Drug effects on perseveration were additionally absent, which was also true when holding the value of the side stickiness parameter constant. The winning model therefore provides the following interpretation of disease and drug effects in this task.

### Dopaminergic modulation of stickiness parameters

We found that amisulpride, but not pramipexole, remediated the elevated stimulus stickiness in SUD, whereas neither drug modulated stimulus stickiness in OCD (Figs. 3, 4 and 5; Tables 4 and 5). This is in contrast to Ersche et al. (2011) who found pramipexole remediated the perseverative deficit in SUD, while their analysis did not detect an effect of amisulpride on behaviour in any group. Both drugs, meanwhile, decreased side (location) stickiness in SUD. These varied pharmacological results are in line with our observation that stimulus stickiness, side (location) stickiness, and perseveration, were distinguishable, yet at times related. Parsing these three conceptually similar measures helps refine the way we study behavioural inflexibility. While the effects of amisulpride and pramipexole on stickiness parameters and perseveration are ostensibly paradoxical, D_2/3_ agonism and antagonism have each produced deficits in reversal learning—on a deterministic schedule in nonhuman animals—and the results are multifaceted. D_2/3_ agonism and antagonism, for instance, affected different aspects of reversal learning (Boulougouris et al. 2009; Lee et al. 2007). D_2/3_ antagonism, additionally, when co-administered with a D_2/3_ agonist, protected against the deficit induced by the agonist, and D_2_ and D_3_ receptors have been shown to play distinct roles in reversal learning (Boulougouris et al. 2009). Although pramipexole may have higher affinity for the D_3_ receptor (Camacho-Ochoa et al. 1995), the respective contributions of D_2_ and D_3_ receptors cannot be dissected using less selective drugs like amisulpride or pramipexole, as in our study. It is also possible that autoreceptor activity, and the doses used, contributed to the directionality of our effects—notions that apply to all of our findings on dopaminergic modulation of computational parameters; Horst et al. (2018), for example, have recently shown that the D_2/3_ agonist quinpirole has triphasic effects on serial deterministic reversal leaning in marmoset monkeys when infused into the caudate nucleus. Performance was impaired at both low and high doses, and improved at intermediate doses; the effects at low doses were likely due to effects of presynaptic autoreceptors. Activation of somato-dendritic autoreceptors versus striatal terminal dopamine autoreceptors, furthermore, may have different effects. Evenden and Robbins (1983) additionally showed an important pattern of results, which raise the notion of baseline-dependency: d-amphetamine induced both response switching and perseveration in the rat. Which behavioural pattern was emitted depended not only on dose—perseveration generally occurred at higher doses—and the task structure, but also on baseline behaviour; the effects of d-amphetamine on behaviour were baseline-dependent. This notion of baseline dependency likely applies to our observation of differential effects of dopaminergic modulation in SUD and OCD; the disorders share important baseline features including abnormal OFC functional connectivity, though do not have an identical neural profile (Meunier et al. 2012).

Understanding the nuances of D_2/3_ receptor involvement in maladaptive behaviour is particularly important given the evidence of reduced striatal D_2_ receptor availability in cocaine abuse (Volkow et al. 1993), methamphetamine abuse (Volkow et al. 2001), and OCD (Denys et al. 2004; Perani et al. 2008; but see Schneier et al. 2008). An analogous pattern has been observed in alcohol (Hietala et al. 1994; Volkow et al. 1996) and opiate dependence (Wang et al. 1997). The studies of cocaine and methamphetamine also tested orbitofrontal cortex (OFC) metabolism, showing an association between lower D_2_ receptor availability and reduced OFC metabolism (Volkow et al. 1993, 2001). Behaviourally, monkeys with greater D_2_-type availability indeed exhibited better performance on a (deterministic) reversal learning task (Groman et al. 2011). At the same time, healthy humans carrying the A1 allele of the dopamine D_2_ receptor Taq1A polymorphism, which is associated with reduced striatal D_2_ receptor expression, showed deficits in PRL (Jocham et al. 2009).

### Perseveration, checking behaviour, and uncertainty

Given SUD and OCD are both disorders of compulsivity and perseveration can be an indicator of compulsivity, it is logical to hypothesise that people with OCD would also show perseveration in this task. Prior studies of OCD, however, have not found evidence of perseveration during PRL (Chamberlain et al. 2007a, b; Ersche et al. 2011; Hauser et al. 2017; Remijnse et al. 2006). Compulsivity is indeed a complex phenomenon and is difficult to capture in a single measure. To that end, we have presented data that enrich our understanding of the multifaceted nature of the construct. Avoiding negative consequences is a key feature of OCD (APA 2013) and is not as central in SUD. This may help explain the divergent task findings in both the conventional perseveration analysis in Ersche et al. (2011) and the computational analyses of stickiness parameters reported here. The probabilistic nature of the task is likely of central importance. The first component of PRL is to learn the optimal behaviour in a stable environment, which requires ignoring rare negative events by not switching choices. Because such probabilistic switching was elevated in our OCD group, we thought it could be related to diminished stimulus stickiness; however, switching was also elevated in SUD. Probabilistic switching (lose-shift), furthermore, was not correlated with stimulus (or side/location) stickiness in SUD or OCD; instead, it was correlated with a higher learning rate for negative feedback in healthy controls, SUD, and OCD, supporting the notion of lose-shifting as a behavioural manifestation of hypersensitivity to negative feedback (Murphy et al. 2003; Taylor Tavares et al. 2008). Moreover, findings from Hauser et al. (2017), who also studied PRL in OCD, are in agreement with our diminished stimulus stickiness result: using a different computational model, individuals with OCD showed a decreased likelihood of repeating the same action, regardless of stimulus value. Hauser et al. (2017), interestingly, did not find elevated probabilistic switching (lose-shift) in their OCD group. Our results in conjunction with Hauser et al. (2017) strengthen the case that diminished stimulus stickiness provides a novel characterization of PRL in OCD. The diminished stimulus stickiness seen in OCD may be a manifestation of checking behaviour, a suggestion also made by Hauser et al. (2017). Checking behaviour, which can be a core symptom in OCD, was indeed modulated by D_2/3_ agents in rats (Eagle et al. 2014), in a translational laboratory model that has also captured increased checking in OCD (Morein-Zamir et al. 2018). Uncertainty is a feature common to both PRL and the translational model of checking behaviour. When reinforcement is deterministic, as opposed to probabilistic, there is less uncertainty and there is no rare event to ignore. Deterministic reversal learning paradigms may consequently be more sensitive to detect perseveration in OCD. Indeed, while serotonin depletion of the OFC in marmosets induced perseveration on a deterministic reversal learning paradigm (Clarke et al. 2004), which appears to reflect behaviour that has become stimulus-bound (Walker et al. 2009), OFC serotonin depletion also impaired PRL but did not induce conventional perseveration (Rygula et al. 2015).

### Reward- and punishment-driven learning

Our computational analysis showed diminished learning from positive feedback in SUD, and an increase in learning from negative feedback in both SUD and OCD (Fig. 3). These results align with the original Ersche et al. (2011) data showing diminished win-stay behaviour (more spontaneous errors) in SUD and increased probabilistic switching (lose-shift) in both SUD and OCD. Ersche et al. (2016), on the other hand, found individuals with SUD were impaired in both reward and avoidance learning, a contrast likely due to several important task differences. Ersche et al. (2016) measured avoidance of electric shock, whereas our task included negative feedback in the form of a sad red face icon, which is notably less salient and presumably engenders less motivation. The appetitive component of each study, however, was more similar in that the positive feedback was given in the form of points or a happy green face icon. Reinforcement, furthermore, was deterministic in the learning phase of the tasks used in Ersche et al. (2016), which removes an element of uncertainty present in probabilistic paradigms. Ersche et al. (2016) measured reward and punishment learning in two separate tasks without reversals, whereas our results here pertain to learning from positive and negative feedback intertwined in the same task—one affected the other. The salience of positive and negative feedback in our task was matched, whereas the salience of the aversive component of Ersche et al. (2016) was greater than the appetitive component. It is possible that the increased learning from negative feedback we observed in SUD was a compensation for the decreased reward learning and increased stimulus stickiness.

How do our learning rate findings in SUD relate to recent data on PRL in alcohol use disorder (AUD)? Reiter et al. (2016) found increased perseveration (albeit calculated differently) and diminished win-stay behaviour in AUD, both present in SUD as reported by Ersche et al. (2011). Their model, like ours, incorporated separate parameters for reward and punishment (nonreward) rates for the chosen stimulus, but they also included parameters for reward and punishment rates that simultaneously tracked the value of the unchosen stimulus. Of these, the only parameter that differed revealed an AUD group deficit in updating the value of the alternative (rewarded) option, following punishment (nonreward) on the chosen stimulus. Whilst this would be interesting to test, it is difficult to extrapolate to our data. At the same time, their lack of an effect on learning rates for the chosen option differs from our findings. We would have expected a diminished reward learning rate in AUD to the chosen option, based on the negative correlation between this parameter and spontaneous errors (increased win-shift) in SUD. Lose-shift behaviour seems to be a key source of divergence in the learning rate results between the two studies: Reiter et al. (2016) did not find a difference in lose-shift between AUD and controls, whereas lose-shift was elevated in our SUD group and correlated positively with the negative feedback learning rate. The lack of lose-shift behaviour in AUD at the outset could be due to task discrepancies—the AUD study used fewer reversals, for instance – or indeed a more general difference in how participants with AUD and SUD learn from negative feedback to guide behaviour in PRL or outside the laboratory.

The increased learning from negative feedback we observed in OCD, meanwhile, is consistent with Gillan et al. (2014) who showed excessive avoidance of electric shock in OCD. We found learning from positive feedback was not different between OCD and control participants, in line with Gillan et al. (2011) who also showed no impairment in reward learning, using a similar task to Ersche et al. (2016). Hauser et al. (2017) reported no difference in learning from reinforcement between healthy controls and OCD on PRL, however their learning rate parameter did not measure positive and negative feedback learning separately, as we report here. Notably, their sample included adolescents with OCD as well as adults, and the neuropsychological profile of OCD in these age groups is not identical (Gottwald et al. 2018). In light of Hauser et al. (2017), we tested a model with a single learning rate and stimulus stickiness parameter (Table 2), and found it ranked third best (Table 3), whereas our winning model was not tested in their analysis. We additionally used a bridge sampling estimate of the marginal likelihood to perform model comparison, which has been newly introduced to practical Bayesian inference (Gronau et al. 2017a, b), and is thought to be an improvement upon the Bayesian information criterion (BIC) as used in Hauser et al. (2017).

### Dopamine and reinforcement learning

Dopamine is well known for its central role in learning about rewards. Nonhuman animal studies have shown that phasic spiking of dopamine neurons signal positive outcomes that are unexpected or more rewarding than anticipated, known as positive prediction errors, whereas the omission of expected reward—negative prediction error—is associated with a reduction of phasic dopaminergic firing (Schultz et al. 1997). Murray et al. (2019), compared the same set of OCD and control participants as in our experiment and found that negative prediction errors were enhanced in OCD and that this was normalised by both amisulpride and pramipexole. Their finding is not only consistent with the role of dopamine in prediction error but also complements our observations that amisulpride and pramipexole produced the same directionality of effects on learning parameters.

In line with the role of dopamine in learning, amisulpride and pramipexole reversed the deficit in learning from positive feedback observed in SUD (Figs. 4b and 5a; Tables 4 and 5), without affecting this parameter in OCD (Figs. 4c and 5b; Tables 4 and 5) or healthy controls (Fig. 4a; Table 5). Negative feedback-driven learning was increased in SUD at baseline and was ameliorated by both drugs (Figs. 4b and 5a; Tables 4 and 5). Both amisulpride and pramipexole increased learning from negative feedback in OCD (Fig. 4c, Table 4) and controls (Fig. 4a; Table 5), and this was the only drug-induced change in healthy individuals detected by our model. This series of results greatly extends the original findings, as Ersche et al. (2011) found no drug effects on spontaneous errors (win-shift) or probabilistic switches (lose-shift). These correlated with the reward and punishment learning rates, respectively, which we show here to be more sensitive to dopaminergic modulation.

Our results on learning rates in SUD in particular, and their dopaminergic modulation, align with work on Parkinson’s disease. Parkinson’s is characterised by dramatic degeneration of dopaminergic neurons in the substantia nigra (Kish et al. 1988) and has therefore been of great importance for understanding dopamine function. SUD (cocaine use), at the same time, is associated with lower levels of endogenous striatal dopamine (Martinez et al. 2009). The mainstay of Parkinson’s treatment is levodopa (L-DOPA), the biosynthetic precursor to dopamine, and is thought to increase phasic dopamine release (Harden and Grace 1995; Pothos et al. 1998). Rutledge et al. (2009) studied individuals with Parkinson’s on and off of L-DOPA, using a dynamic foraging task with probabilistic and reversal elements. They employed computational modelling and showed L-DOPA increased learning rates to rewards and remediated perseverative deficits. Both of these findings are consistent with our results in SUD on reward learning rates, stimulus stickiness, and their dopaminergic modulation. The perseveration parameter in Rutledge et al. (2009), like our stimulus stickiness measure, was independent of reward history. Our results on positive and negative learning rates in SUD are also consistent with conventional analyses by Frank et al. (2004): Individuals with Parkinson’s, when off medication, were better at learning from negative feedback, as assessed by probabilistic and deterministic tasks without reversals (Frank et al. 2004). When a reward is omitted, there is ordinarily a dip in dopaminergic firing (Schultz et al. 1997), and in the setting of dopamine depletion in Parkinson’s, this mechanism of learning from negative feedback appears to be facilitated, at least when assessed using outcomes of points or money (Frank et al. 2004). On L-DOPA reward learning was enhanced, consistent with Rutledge et al. (2009), and the elevated learning from negative feedback was normalised (Frank et al. 2004).

Eisenegger et al. (2014) showed a single 800 mg dose of the D_2/3_ antagonist sulpiride impaired choice performance for probabilistic rewards without affecting responses to punishment in healthy male volunteers—no females were studied. Superficially, the result from Eisenegger et al. (2014) may seem at odds with our reward and punishment learning findings; however, several key differences between their experiment and ours appear to account for the discrepancy. While their task was also probabilistic, there were no reversals, and they tested appetitive and aversive learning in separate blocks (gain of money versus nil, or loss of money versus nil). Sulpiride and amisulpride are similar; both are from the benzamine class of atypical antipsychotics. For the treatment of psychosis, a normal dose of amisulpride is 400–800 mg per day, whereas the range for sulpiride is 400–2400 mg per day (www.medicines.org.uk). Critically, the higher dose used in their study could have led to greater striatal D_2_ occupancy. Indeed, a single 800 mg dose of sulpiride has been reported to occupy ~ 60% of striatal dopamine D_2_ receptors (Takano et al. 2006), whereas a single 400 mg dose of sulpiride occupies ~ 30% in healthy volunteers (Mehta et al. 2008). This is especially important because the pharmacological effects in Eisenegger et al. (2014) were driven by participants who achieved higher blood levels of sulpiride and by individuals with genetic variation associated with diminished D_2_ receptor expression. Presumably those with lower D_2_ receptor expression are disproportionately sensitive to D_2/3_ modulation. In fact, they found no effects on their classical or Bayesian analyses for participants with a low blood sulpiride level, determined by median split. Using classical statistics, Eisenegger et al. (2014) reported a selective impairment on the reward but not punishment component; however, this was limited to a late phase after learning had reached asymptote, suggesting behavioural expression rather than learning was disrupted; this complicates comparison with PRL. In line with this, in a Bayesian analysis, they found sulpiride increased a temperature parameter in the appetitive domain only, which reflects increased choice switching. Eisenegger et al. (2014) also found sulpiride did not affect the learning rate in their appetitive or aversive component, regardless of blood levels or genetics; task design again precludes meaningful comparison. Finally, because their genetic data on the D_2_ receptor and their effects of a nonselective D_2/3_ agent aligned, they were able to infer their results were driven by D_2_ modulation, which was not possible in our study. Differences in the task contingencies and feedback structure between our task and theirs, the drug dose, and variation of D_2_ receptor density likely account for the discrepancies between results.

### Consideration of medication status

It is important to note that most of those in the OCD group were medicated with SSRIs, which are known to affect dopaminergic signalling (Pogarell et al. 2005) and also modulate PRL. A single dose of an SSRI, more specifically, has promoted hypersensitivity to negative feedback in rats and healthy humans, and subchronic dosing has improved performance in rats (Bari et al. 2010; Chamberlain et al. 2006; Skandali et al. 2018). The hypersensitivity to negative feedback seen in unmedicated depression (Taylor Tavares et al. 2008) was also present in depressed individuals treated with SSRIs (Murphy et al. 2003), and PRL deficits in OCD persist despite SSRI use as well (Ersche et al. 2011; Hauser et al. 2017; Remijnse et al. 2006). A possible explanation is that SSRIs do not modulate serotonergic activity in the OFC as readily as in other parts of the frontal cortex (El Mansari et al. 1995), and OFC abnormalities are present in depression (Bremner et al. 2002), as well as in OCD.

### Implications for treatment

Our results are important for informing and refining treatment approaches. While there have been no randomised placebo controlled clinical trials of amisulpride for the treatment of OCD, other D_2/3_ antagonists are often used as an effective augmentation of first-line SSRI therapy in treatment resistant cases of OCD (Fineberg et al. 2013). At the same time, no studies have assessed the clinical efficacy of the D_2/3_ agonist pramipexole for OCD. There have been numerous studies testing dopaminergic agonists for the treatment of SUD; however, evidence for their clinical efficacy is currently lacking (Minozzi et al. 2015). To our knowledge, there has been no clinical trial of amisulpride for the treatment of SUD. Existing studies testing other D_2/3_ antagonists have mostly found a lack of clinical benefit for SUD, or even a worsening of symptoms; however, multiple studies have found D_2/3_ antagonists to reduce cravings in those with comorbid psychosis (Zhornitsky et al. 2010).

### Conclusion

We have, to our knowledge, conducted the first comparison of the computational processes underlying two disorders of compulsivity, and their dopaminergic modulation. We have shown that an RL model captured mechanisms that differed between individuals with SUD, OCD, and healthy controls, and also detected changes following dopaminergic drug administration. The parameters in our model revealed subtleties underlying maladaptive behaviour that considerably extend conventional analyses of PRL. One key novel finding was that the stimulus stickiness parameter differentiated all three groups, with opposing effects in SUD and OCD. Behaviour in SUD was driven primarily by a combination of increased stimulus stickiness and an imbalance of learning from positive versus negative feedback—decreased positive and increased negative feedback learning. The altered computations underlying performance in OCD, on the other hand, were a decrease in stimulus stickiness and an increase in learning from negative feedback. D_2/3_ modulation normalised the stimulus stickiness anomalies in SUD, in particular, and reversed deficits in other parameters as well. Our computational analysis allowed for a more nuanced cross-species comparison of the neural basis of PRL, with implications for its neurochemical modulation. The results, taken in the context of the existing literature, highlight the importance of considering how drug dose, receptor subtypes and expression, clinical phenotype, and subtleties of the task environment—the salience of feedback, deterministic or probabilistic reinforcement, single or serial reversals, and the role of uncertainty—may interact to affect behaviour and its underlying computational structure. By using Bayesian hierarchical modelling, we can begin to understand the subtle mechanisms that contribute to maladaptive responses on tests of behavioural flexibility, and their neurochemical basis in health and disease. This may eventually inform susceptibility to illness, diagnosis, and treatment.

## Electronic supplementary material

ESM 1 (R 22 kb)

ESM 2 (R 35 kb)

ESM 3 (STAN 2 kb)

ESM 4 (STAN 1 kb)

ESM 5 (PY 5 kb)

ESM 6 (CSV 5743 kb)

ESM 7 (M 7 kb)

ESM 8 (STAN 22 kb)

ESM 9 (STAN 27 kb)

ESM 10 (STAN 28 kb)

ESM 11 (STAN 22 kb)

ESM 12 (STAN 18 kb)

ESM 13 (STAN 19 kb)

ESM 14 (STAN 15 kb)

ESM 15 (STAN 14 kb)

ESM 16 (PY 39 kb)

ESM 17 (PY 1 kb)

ESM 18 (PY 1 kb)

ESM 19 (STAN 161 kb)

ESM 20 (R 1 kb)

ESM 21 (R 1 kb)

ESM 22 (PY 79 kb)

ESM 23 (R 3 kb)

ESM 24 (R 4 kb)

ESM 25 (R 43 kb)

ESM 26 (R 12 kb)

ESM 27 (R 60 kb)

ESM 28 (DOCX 66 kb)

## References

[CR1] American Psychiatric
Association (2000). Diagnostic and statistical manual of mental disorders, 4th edition,
Text Revision.

[CR2] American Psychiatric Association (2013) Diagnostic and statistical manual of mental disorders, 5th edition. American Psychiatric Association, Washington, DC

[CR3] Bari A, Theobald DE, Caprioli D, Mar AC, Aidoo-Micah A, Dalley JW, Robbins TW (2010). Serotonin modulates sensitivity to reward and negative
feedback in a probabilistic reversal learning task in rats. Neuropsychopharmacology.

[CR4] Beck AT, Steer RA, Brown GK (1996). Manual for Beck depression inventory—II.

[CR5] Boulougouris V, Castañé A, Robbins TW (2009). Dopamine D2/D3 receptor agonist quinpirole impairs
spatial reversal learning in rats: investigation of D3 receptor involvement in
persistent behavior. Psychopharmacology.

[CR6] Bremner JD, Vythilingam M, Vermetten E, Nazeer A, Adil J, Khan S, Staib LH, Charney DS (2002). Reduced volume of orbitofrontal cortex in major
depression. Biol Psychiatry.

[CR7] Brooks SP, Gelman A (1998). General methods for monitoring convergence of
iterative simulations. J Comput Graph Stat.

[CR8] Camacho-Ochoa M, Walker EL, Evans DL, Piercey MF (1995). Rat brain binding sites for pramipexole, a clinically
useful D3-preferring dopamine agonist. Neurosci Lett.

[CR9] Camerer C, Ho TH (1999). Experience-weighted attraction learning in normal form
games. Econometrica.

[CR10] Carpenter B, Gelman A, Hoffman MD, Lee D, Goodrich B, Betancourt M, Brubaker M, Guo J, Li P, Riddell A (2017). Stan: a probabilistic programming
language. J Stat Softw.

[CR11] Chamberlain SR, Müller U, Blackwell AD (2006). Neurochemical modulation of response inhibition and
probabilistic learning in humans. Science.

[CR12] Chamberlain SR, Fineberg NA, Blackwell AD, Clark L, Robbins TW, Sahakian BJ (2007). A neuropsychological comparison of
obsessive-compulsive disorder and trichotillomania. Neuropsychologia.

[CR13] Chamberlain SR, Fineberg NA, Menzies LA (2007). Impaired cognitive flexibility and motor inhibition in
unaffected first-degree relatives of patients with obsessive-compulsive
disorder. Am J Psychiatry.

[CR14] Christakou A, Gershman SJ, Niv Y, Simmons A, Brammer MRK (2013). Neural and psychological maturation of decision-making
in adolescence and young adulthood. J Cogn Neurosci.

[CR15] Clarke HF, Dalley JW, Crofts HS, Robbins TW, Roberts AC (2004) Cognitive inflexibility after prefrontal serotonin depletion. Science 304:878–880. 10.1126/science.1094987 10.1126/science.1094987 15131308

[CR16] Clarke HF, Cardinal RN, Rygula R, Hong YT, Fryer TD, Sawiak SJ, Ferrari V, Cockcroft G, Aigbirhio FI, Robbins TW, Roberts AC (2014). Orbitofrontal Dopamine Depletion Upregulates Caudate
Dopamine and Alters Behavior via Changes in Reinforcement
Sensitivity. J Neurosci.

[CR17] Daw ND, Delgado MR, Phelps EA, Robbins TW (2011). Trial-by-trial data analysis using computational
models. Decision making, affect, and learning: attention and performance
XXIII.

[CR18] Denys D, Van Der Wee N, Janssen J (2004). Low level of dopaminergic D2 receptor binding in
obsessive-compulsive disorder. Biol Psychiatry.

[CR19] Eagle DM, Noschang C, d’Angelo L-SC (2014). The dopamine D2/D3 receptor agonist quinpirole
increases checking-like behaviour in an operant observing response task with
uncertain reinforcement: a novel possible model of OCD. Behav Brain Res.

[CR20] Eisenegger C, Naef M, Linssen A, Clark L, Gandamaneni PK, Müller U, Robbins TW (2014). Role of dopamine D2 receptors in human reinforcement
learning. Neuropsychopharmacology.

[CR21] Ersche KD, Roiser JP, Robbins TW, Sahakian BJ (2008). Chronic cocaine but not chronic amphetamine use is
associated with perseverative responding in humans. Psychopharmacology.

[CR22] Ersche KD, Roiser JP, Abbott S, Craig KJ, Müller U, Suckling J, Ooi C, Shabbir SS, Clark L, Sahakian BJ, Fineberg NA, Merlo-Pich EV, Robbins TW, Bullmore ET (2011). Response perseveration in stimulant dependence is
associated with striatal dysfunction and can be ameliorated by a D2/3 receptor
agonist. Biol Psychiatry.

[CR23] Ersche KD, Gillan CM, Jones PS, et al (2016) Carrots and sticks fail to change behavior in cocaine addiction. 352:1468–1471. 10.1126/science.aaf3700 10.1126/science.aaf3700 PMC5144994 27313048

[CR24] Evenden JL, Robbins TW (1983). Increased response switching, perseveration and
perseverative switching following d-amphetamine in the rat. Psychopharmacology.

[CR25] Everitt BJ, Robbins TW (2016). Drug addiction: updating actions to habits to
compulsions ten years on. Annu Rev Psychol.

[CR26] Fineberg NA, Reghunandanan S, Brown A, Pampaloni I (2013) Pharmacotherapy of obsessive-compulsive disorder: evidence-based treatment and beyond. Aust N Z J Psychiatry 47:121-141. 10.1177/0004867412461958 10.1177/0004867412461958 23125399

[CR27] First MB, Spitzer RL, Gibbon M, Williams JBW (2002). Structured clinical interview for DSM-IV-TR Axis I disorders, research
version, non-patient edition. (SCID-I/NP).

[CR28] Frank MJ, Seeberger LC, O’Reilly RC (2004). By carrot or by stick: cognitive reinforcement
learning in parkinsonism. Science.

[CR29] Franken IHA, Hendriks VM, van den Brink W (2002). Initial validation of two opiate craving
questionnaires. Addict Behav.

[CR30] Gelman A, Tuerlinckx F (2000). Type S error rates for classical and Bayesian single
and multiple comparison procedures 1 Introduction. Comput Stat.

[CR31] Gelman A, Hill J, Yajima M (2012). Why we (usually) don’t have to worry about
multiple. J Res Educ Eff.

[CR32] Gelman A, Carlin JB, Stern HS, Dunson DB, Vehtari A, Rubin DB (2013). Bayesian data analysis.

[CR33] Gershman SJ (2016). Empirical priors for reinforcement learning
models. J Math Psychol.

[CR34] Gillan CM, Papmeyer M, Morein-Zamir S, Sahakian BJ, Fineberg NA, Robbins TW, de Wit S (2011). Disruption in the balance between goal-directed
behavior and habit learning in obsessive-compulsive disorder. Am J Psychiatry.

[CR35] Gillan CM, Morein-Zamir S, Urcelay GP, Sule A, Voon V, Apergis-Schoute AM, Fineberg NA, Sahakian BJ, Robbins TW (2014). Enhanced avoidance habits in obsessive-compulsive
disorder. Biol Psychiatry.

[CR36] Goodman WK, Price LH, Rasmussen SA, Mazure C, Fleischmann RL, Hill CL, Heninger GR, Charney DS (1989). The Yale-Brown Obsessive Compulsive Scale. I.
Development, use, and reliability. Arch Gen Psychiatry.

[CR37] Gottwald J, De Wit S, Apergis-Schoute AM (2018). Impaired cognitive plasticity and goal-directed
control in adolescent obsessive-compulsive disorder. Psychol Med.

[CR38] Groman SM, Lee B, London ED, Mandelkern MA, James AS, Feiler K, Rivera R, Dahlbom M, Sossi V, Vandervoort E, Jentsch JD (2011). Dorsal striatal D2-like receptor availability covaries
with sensitivity to positive reinforcement during discrimination
learning. J Neurosci.

[CR39] Gronau QF, Sarafoglou A, Matzke D, Ly A, Boehm U, Marsman M, Leslie DS, Forster JJ, Wagenmakers EJ, Steingroever H (2017). A tutorial on bridge sampling. J Math Psychol.

[CR40] Gronau QF, Singmann H, Wagenmakers E-J (2017b) Bridgesampling: an R package for estimating normalizing constants. ArXiv 1710.08162 [stat.CO]. http://arxiv.org/abs/1710.08162

[CR41] Harden DG, Grace A (1995). Activation of dopamine cell firing by repeated L-DOPA
administration to dopamine-depleted rats: its potential role in mediating the
therapeutic response to L-DOPA treatment. J Neurosci.

[CR42] Hauser TU, Iannaccone R, Dolan RJ, Ball J, Hättenschwiler J, Drechsler R, Rufer M, Brandeis D, Walitza S, Brem S (2017). Increased fronto-striatal reward prediction errors
moderate decision making in obsessive-compulsive disorder. Psychol Med.

[CR43] Hietala J, West C, Syvälahti E, Någren K, Lehikoinen P, Sonninen P, Ruotsalainen U (1994). Striatal D2 dopamine receptor binding characteristics
in vivo in patients with alcohol dependence. Psychopharmacology.

[CR44] Horst NK, Jupp B, Roberts AC, Robbins TW (2019). D2 receptors and cognitive flexibility in marmosets:
tri-phasic dose–response effects of intra-striatal quinpirole on serial reversal
performance. Neuropsychopharmacology.

[CR45] Jocham G, Klein TA, Neumann J, von Cramon DY, Reuter M, Ullsperger M (2009). Dopamine DRD2 polymorphism alters reversal learning
and associated neural activity. J Neurosci.

[CR46] Kish SJ, Shannak K, Hornykiewicz O (1988). Uneven pattern of dopamine loss in the striatum of
patients with idiopathic Parkinson’s disease. N Engl J Med.

[CR47] Kruschke JK (2011). Bayesian assessment of null values via parameter
estimation and model comparison. Perspect Psychol Sci.

[CR48] Kruschke JK (2011). Doing Bayesian Data Analysis.

[CR49] Lawrence AD, Sahakian BJ, Rogers RD, Hodges JR, Robbins TW (1999). Discrimination, reversal, and shift learning in
Huntington’s disease: mechanisms of impaired response selection. Neuropsychologia.

[CR50] Lee B, Groman S, London ED, Jentsch JD (2007). Dopamine D2/D3 receptors play a specific role in the
reversal of a learned visual discrimination in monkeys. Neuropsychopharmacology.

[CR51] MacKay DJC (2003). Information theory, inference, and learning algorithms.

[CR52] el Mansari M, Bouchard C, Blier P (1995). Alteration of serotonin release in the guinea pig
orbito-frontal cortex by selective serotonin reuptake inhibitors. Relevance to
treatment of obsessive-compulsive disorder. Neuropsychopharmacology.

[CR53] Martinez D, Greene K, Broft A (2009). Lower level of endogenous dopamine in patients with
cocaine dependence: findings from PET imaging of D2/D3 receptors following acute
dopamine depletion. Am J Psychiatry.

[CR54] Mehta MA, Montgomery AJ, Kitamura Y, Grasby PM (2008). Dopamine D2 receptor occupancy levels of acute
sulpiride challenges that produce working memory and learning impairments in
healthy volunteers. Psychopharmacology.

[CR55] Meunier D, Ersche KD, Craig KJ, Fornito A, Merlo-Pich E, Fineberg NA, Shabbir SS, Robbins TW, Bullmore ET (2012). Brain functional connectivity in stimulant drug
dependence and obsessive-compulsive disorder. Neuroimage.

[CR56] Minozzi S, Amato L, Pani PP, Solimini R, Vecchi S, De Crescenzo F, Zuccaro P, Davoli M (2015) Dopamine agonists for the treatment of cocaine dependence. Cochrane Database Syst Rev 5:CD003352. 10.1002/14651858.CD003352.pub4 10.1002/14651858.CD003352.pub4 PMC6999795 26014366

[CR57] Mkrtchian A, Aylward J, Dayan P, Roiser JP, Robinson OJ (2017). Modeling avoidance in mood and anxiety disorders using
reinforcement learning. Biol Psychiatry.

[CR58] Morein-Zamir S, Shahper S, Fineberg NA, Eisele V, Eagle DM, Urcelay G, Robbins TW (2018). Free operant observing in humans: a translational
approach to compulsive certainty seeking. Q J Exp Psychol (Hove).

[CR59] Murphy FC, Michael A, Robbins TW, Sahakian BJ (2003). Neuropsychological impairment in patients with major
depressive disorder: the effects of feedback on task performance. Psychol Med.

[CR60] Murray GK, Knolle F, Ersche KD, Craig KJ, Abbott A, Shabbir SS, Fineberg NA, Suckling J, Sahakian BJ, Bullmore ET, Robbins TW (2019) Dopaminergic drug treatment remediates exaggerated cingulate prediction error responses in obsessive-compulsive disorder. Psychopharmacology. 10.1007/s00213-019-05292-2 10.1007/s00213-019-05292-2 PMC6695357 31201476

[CR61] den Ouden HEM, Daw ND, Fernandez G, Elshout JA, Rijpkema M, Hoogman M, Franke B, Cools R (2013). Dissociable effects of dopamine and serotonin on
reversal learning. Neuron.

[CR62] Patton JH, Stanford MS, Barratt ES (1995). Factor structure of the Barratt impulsiveness
scale. J Clin Psychol.

[CR63] Perani D, Garibotto V, Gorini A, Moresco RM, Henin M, Panzacchi A, Matarrese M, Carpinelli A, Bellodi L, Fazio F (2008). In vivo PET study of 5HT2A serotonin and D2 dopamine
dysfunction in drug-naive obsessive-compulsive disorder. Neuroimage.

[CR64] Pogarell O, Poepperl G, Mulert C, Hamann C, Sadowsky N, Riedel M, Moeller HJ, Hegerl U, Tatsch K (2005). SERT and DAT availabilities under citalopram treatment
in obsessive-compulsive disorder (OCD). Eur Neuropsychopharmacol.

[CR65] Pothos EN, Davila V, Sulzer D (1998). Presynaptic recording of quanta from midbrain dopamine
neurons and modulation of the quantal size. J Neurosci.

[CR66] Reiter AMF, Deserno L, Kallert T, Heinze HJ, Heinz A, Schlagenhauf F (2016). Behavioral and neural signatures of reduced updating
of alternative options in alcohol-dependent patients during flexible
decision-making. J Neurosci.

[CR67] Remijnse PL, Nielen MMA, van Balkom AJLM, Cath DC, van Oppen P, Uylings HBM, Veltman DJ (2006). Reduced orbitofrontal-striatal activity on a reversal
learning task in obsessive-compulsive disorder. Arch Gen Psychiatry.

[CR68] Rutledge RB, Lazzaro SC, Lau B, Myers CE, Gluck MA, Glimcher PW (2009). Dopaminergic drugs modulate learning rates and
perseveration in Parkinson’s patients in a dynamic foraging task. J Neurosci.

[CR69] Rygula R, Clarke HF, Cardinal RN, Cockcroft GJ, Xia J, Dalley JW, Robbins TW, Roberts AC (2015). Role of central serotonin in anticipation of rewarding
and punishing outcomes: effects of selective amygdala or orbitofrontal 5-HT
depletion. Cereb Cortex.

[CR70] Schneier FR, Martinez D, Abi-Dargham A, Zea-Ponce Y, Simpson HB, Liebowitz MR, Laruelle M (2008) Striatal dopamine D2 receptor availability in OCD with and without comorbid social anxiety disorder: preliminary findings. Depress Anxiety 25:1–7. https://doi.org/10.1002/da 10.1002/da.20268 17252580

[CR71] Schultz W, Dayan P, Montague PR (1997). A neural substrate of prediction and
reward. Science.

[CR72] Seymour B, Daw ND, Roiser JP, Dayan P, Dolan R (2012). Serotonin selectively modulates reward value in human
decision-making. J Neurosci.

[CR73] Skandali N, Rowe JB, Voon V, Deakin JB, Cardinal RN, Cormack F, Passamonti L, Bevan-Jones WR, Regenthal R, Chamberlain SR, Robbins TW, Sahakian BJ (2018). Dissociable effects of acute SSRI (escitalopram) on
executive, learning and emotional functions in healthy humans. Neuropsychopharmacology.

[CR74] Takano A, Suhara T, Yasuno F, Suzuki K, Takahashi H, Morimoto T, Lee YJ, Kusuhara H, Sugiyama Y, Okubo Y (2006). The antipsychotic sultopride is overdosed - a PET
study of drug-induced receptor occupancy in comparison with
sulpiride. Int J Neuropsychopharmacol.

[CR75] Taylor Tavares JV, Clark L, Furey ML, Williams GB, Sahakian BJ, Drevets WC (2008). Neural basis of abnormal response to negative feedback
in unmedicated mood disorders. Neuroimage.

[CR76] Volkow ND, Fowler JS, Wang G-J, Hitzemann R, Logan J, Schlyer DJ, Dewey SL, Wolf AP (1993). Decreased dopamine D2 receptor availability is
associated with reduced frontal metabolism in cocaine abusers. Synapse.

[CR77] Volkow ND, Wang GJ, Fowler JS, Logan J, Hitzemann R, Ding YS, Pappas N, Shea C, Piscani K (1996). Decreases in dopamine receptors but not in dopamine
transporters in alcoholics. Alcohol Clin Exp Res.

[CR78] Volkow ND, Chang L, Wang G-J, Fowler JS, Ding YS, Sedler M, Logan J, Franceschi D, Gatley J, Hitzemann R, Gifford A, Wong C, Pappas N (2001). Low level of brain dopamine D2 receptors in
methamphetamine abusers: association with metabolism in the orbitofrontal
cortex. Am J Psychiatry.

[CR79] Walker SC, Robbins TW, Roberts AC (2009). Differential contributions of dopamine and serotonin
to orbitofrontal cortex function in the marmoset. Cereb Cortex.

[CR80] Wang GJ, Volkow ND, Fowler JS, Logan J, Abumrad NN, Hitzemann RJ, Pappas NS, Pascani K (1997). Dopamine D2 receptor availability in opiate-dependent
subjects before and after naloxone-precipitated withdrawal. Neuropsychopharmacology.

[CR81] Wilson RC, Collins C (2019) Ten simple rules for the computational modeling of behavioral data. Preprint PsyArXiv 10.31234/osf.io/46mbn 10.7554/eLife.49547 PMC6879303 31769410

[CR82] Zhornitsky S, Rizkallah E, Pampoulova T, Chiasson JP, Stip E, Rompré PP, Potvin S (2010). Antipsychotic agents for the treatment of substance
use disorders in patients with and without comorbid psychosis. J Clin Psychopharmacol.

