# Supplementary material for: Computational modelling reveals contrasting effects on reinforcement learning and cognitive flexibility in stimulant use disorder and obsessive-compulsive disorder: remediating effects of dopaminergic D2/3 receptor agents
Source: Psychopharmacology (Berl). 2019 Jul 20;236(8):2337–58. doi: 10.1007/s00213-019-05325-w (PMC6820481; doi:10.1007/s00213-019-05325-w)
Supplement: Supplementary file 28 — (DOCX 66 kb) [file 213_2019_5325_MOESM28_ESM.docx]

*SUPPLEMENTARY MATERIAL*

*For Special Issue of Psychopharmacology*

*on Translational Computational Psychopharmacology*

**Computational modelling reveals contrasting effects on reinforcement learning and cognitive flexibility in stimulant use disorder and obsessive-compulsive disorder: Remediating effects of dopaminergic D2/3 receptor agents**

Jonathan W. Kanen^1,2,7^, Karen D. Ersche^2,3^, Naomi A. Fineberg^3,5,6^, Trevor W. Robbins^1,2^,

Rudolf N. Cardinal^2,3,4^

^1^Department of Psychology, University of Cambridge

^2^Behavioural and Clinical Neuroscience Institute, University of Cambridge

^3^Department of Psychiatry, University of Cambridge

^4^Cambridgeshire and Peterborough NHS Foundation Trust

^5^Hertfordshire Partnership University NHS Foundation Trust, Welwyn Garden City, Hertfordshire, United Kingdom

^6^University of Hertfordshire, Department of Postgraduate Medicine, College Lane Hatfield, United Kingdom

^7^Correspondence can be addressed to jonathan.kanen@gmail.com

**Methods:**

MODEL 3

This model includes a reward rate, a punishment rate, and a parameter governing the effect of reinforcement learning upon choice. It is equivalent to the “RP” model from den Ouden et al. (2013). In notation compatible with our manuscript, the value of the chosen stimulus (only) is updated according to

*V_i,t_*_+1_ ← *V_i,t_* + *α^rew^*(*R_t_* – *V_i,t_*) for rewarded trials (*R_t_* = 1)

*V_i,t_*_+1_ ← *V_i,t_* + *α^pun^*(*R_t_* – *V_i,t_*) for punished trials (*R_t_* = 0)

$$P\left( \text{choose stimulus}i\text{on trial}t \right)=\frac{e^{\tau^{reinf}V_{i,t}}}{\sum_{\text{k=}1}^{n} e^{\tau^{reinf}V_{k,t}}}$$

The model’s parameters are *α^rew^*, *α^pun^*, and *τ^reinf^*.

MODEL 5

This model is the experience-weighted attraction (EWA) model of den Ouden et al. (2013), itself after Camerer & Ho (1999). It has three parameters: *φ* (phi)*, ρ* (rho), and *β* (beta).

The “experience weight” of a choice or stimulus that is chosen, *n_c,t_*, is updated according to the experience decay factor *ρ* (range 0 < *ρ* < 1) (den Ouden et al. 2013, equation 1, after Camerer & Ho 1999, equation 2.1):

*n_c,t_* ← *n_c,t_*_–1_ *ρ* + 1

The experience weight is a measure of how often the subject has experienced the action (e.g. chosen a stimulus) – the “number of ‘observation-equivalents’ of past experience” (Camerer & Ho 1999). It can increase unbounded.

The value of a choice is updated according to the outcome, *λ*, and the decay factor for previous payoffs, *φ* (range 0 < *φ* < 1) (den Ouden et al. 2013, equation 2; compare Camerer & Ho 1999, equation 2.2, who use capital Φ in place of lower-case *φ*, and have a more complex payoff term involving π):

*v_c,t_* ← (*v_c,t_*_–1_ *φ* *n_c,t_*_–1_ + *λ_t_*_–1_) / *n_c,t_*

The payoff decay factor *φ* (phi) is related to a Rescorla–Wagner-style (Rescorla and Wagner, 1972) learning rate *α*, by *α* = 1 – *φ*. A high value of *φ* means that stimuli keep a high fraction of their previous value (slow learning from reinforcement).

When *ρ* is high, then “well-known” actions (with high *n*) are updated relatively little by reinforcement, by virtue of the terms involving *n*, while reinforcement has a proportionately larger effect on novel actions (with low *n*). When *ρ* = 0, *n* is always 1 and the value update rule reduces to

| *v_c,t_* ← | *v_c,t_*_–1_ *φ* + *λ_t_*_–1_ |
| --- | --- |
| Δv | *= v_c,t_*_–1_ *φ* + *λ_t_*_–1_ – *v_c,t_*_–1_ |
|  | = (*φ* – 1)*v_c,t_*_–1_ + *λ_t_*_–1_  = *λ_t_*_–1_  – (1 – *φ*)*v_c,t_*_–1_ |
| which, for constant reinforcement, has its asymptote at *v* = *λ/*(1 – *φ*) = *λ*/*α*. | |
| Assigning α = 1 – φ, |  |
| Δv | *= λ_t_*_–1_ – *α* *v_c,t_*_–1_  *= α*(*λ_t_*_–1_/*α* – *v_c,t_*_–1_) |
| making the equivalence clear to the Rescorla–Wagner rule: | |
| ΔV | *= αβ*(*λ* – V) |

In the EWA model, choice is governed by a softmax process, where *β* is a softmax inverse temperature (den Ouden et al. 2013, equation 5):

$$P\left( c_{t+1}=i \right)=\frac{e^{\beta Q\left( c=i,t+1 \right)}}{\sum_{j} e^{\beta Q\left( c=j,t+1 \right)}}$$

where, in this instance, the action value *Q_c,t_* is simply the stimulus value *v_c,t_.*

In our study, we applied the constraint *β* > 0 (since negative values of *β* imply that the preferred choice is less likely to be chosen than others, contrary to the definition of preference). Values of *n* and *v* began at 0. We updated *n* only for stimuli that were chosen. We used *λ* = 1 for reward and *λ* = 0 for nonreward.

SIMULATED PROBABILISTIC REVERSAL LEARNING TASK

As described in the Methods, we simulated virtual subjects in the probabilistic reversal learning task of Ersche et al. (2011). Subjects were presented with a pair of stimuli in a two-stimulus discrimination learning task for 10 “blocks” (learning followed by 9 consecutive reversals with the same stimuli), followed by another (novel) pair of stimuli for a further 10 blocks. The assignment of correct/incorrect stimulus to left/right side was random. The probability of reward given a correct response was 0.85. The probability of reward given an incorrect response was 0. Subjects were deemed to have completed a block when they had performed a certain number of correct responses within that block; this criterion was randomly drawn for each block from the range [10, 15], with the constraint that the criterion was never the same for two consecutive blocks.

**Results:**

*Parameter recovery from the winning model*

We verified parameter recovery from the winning model. The Bayesian method we used for analysis is computationally intensive and verification of 100 values per parameter, as suggested by Wilson & Collins (2019), was not feasible on our high-performance computing cluster (whether combinatorially, varying one parameter at a time, or as 100 sets of 5-tuples), even without the simulation of group/drug effects. We therefore used 5 values per parameter, spanning a range that exceeded the posterior group mean values from the actual data set (with the addition also of τ^reinf^ = 1 for debugging purposes). We set all parameters but one to the central value and varied one parameter at a time, as shown in Table 1 below. For each run, we simulated 100 identical virtual subjects in a single virtual group performing the probabilistic reversal learning task (as described earlier). We analysed the simulated data exactly as we analysed the real data, using the winning Bayesian hierarchical model and with the same priors, except we removed the experimental (group/drug) structure and inter-subject variability (since there was none) to leave the core RL-based algorithm. For each parameter, we measured the mean of its posterior distribution. Results are presented in Table 1 below.

**Additional references:**

Rescorla RA, Wagner AR (1972) A theory of Pavlovian conditioning: variations in the effectiveness of reinforcement and nonreinforcement. In Black AH, Prokasy WF (eds) Classical Conditioning II: Current Research and Theory. Appleton-Century-Crofts, New York, pp 64-99

**Table 1:** parameter recovery by the winning RL model from simulated data (see text). 100 virtual subjects were simulated for each row, with the parameters shown, and the parameters estimated from the resulting data via a hierarchical Bayesian model as described in the text. Recovered parameters are shown in the format “m [a, b] (R=$\hat{R}$)” where m is the posterior mean, [a, b] is the 95% HDI, and $\hat{R}$ is the potential scale factor reduction measure of convergence. † indicates values for which the parameters were not correctly recovered as judged by the 95% HDI (occurring in 2/110 tests, for which the true value was slightly outside the 95% HDI, though inside the 99% HDI in both cases).

| Param. | *α^rew^* | | *α^pun^* | | *τ^reinf^* | | *τ^loc^* | | *τ^stim^* | |
| --- | --- | --- | --- | --- | --- | --- | --- | --- | --- | --- |
| Sim.# | Sim. | Recovered | Sim. | Recovered | Sim. | Recovered | Sim. | Recovered | Sim. | Recovered |
| 1 | **0.1** | 0.101 [0.084, 0.118] (R=1.002) | **0.5** | 0.502 [0.482, 0.522] (R=1.000) | **4.5** | 4.334 [3.826, 4.968] (R=1.002) | **0** | 0.005 [–0.019, 0.030] (R=1.000) | **0** | 0.006 [–0.022, 0.034] (R=1.000) |
| 2 | **0.3** | 0.295 [0.274, 0.316] (R=1.001) | ” | 0.511 [0.493, 0.528] (R=1.000) | ” | 4.528 [4.338, 4.728] (R=1.001) | ” | –0.008 [–0.039, 0.025] (R=1.001) | ” | 0.002 [–0.038, 0.042] (R=1.000) |
| 3 | **0.5** | 0.480 [0.453, 0.508] (R=1.001) | ” | 0.507 [0.490, 0.523] (R=1.000) | ” | 4.600 [4.451, 4.757] (R=1.001) | ” | 0.027 [–0.009, 0.063] (R=1.000) | ” | –0.013 [–0.060, 0.034] (R=1.001) |
| 4 | **0.7** | 0.707 [0.672, 0.743] (R=1.001) | ” | 0.492 [0.476, 0.509] (R=1.000) | ” | 4.444 [4.320, 4.576] (R=1.000) | ” | –0.031 [–0.069, 0.009] (R=1.000) | ” | –0.009 [–0.058, 0.041] (R=1.000) |
| 5 | **0.9** | 0.906 [0.862, 0.951] (R=1.000) | ” | 0.490 [0.474, 0.507] (R=1.000) | ” | 4.517 [4.387, 4.648] (R=1.000) | ” | –0.015 [–0.054, 0.025] (R=1.000) | ” | –0.031 [–0.083, 0.021] (R=1.000) |
| 6 | 0.5 | 0.497 [0.476, 0.519] (R=1.000) | **0.1** | 0.099 [0.095, 0.104] (R=1.000) | ” | 4.416 [4.277, 4.559] (R=0.999) | ” | –0.006 [–0.034, 0.022] (R=1.000) | ” | **† 0.041 [0.008, 0.075] (R=1.000)** |
| 7 | ” | 0.502 [0.477, 0.528] (R=1.001) | **0.3** | 0.297 [0.284, 0.310] (R=1.001) | ” | 4.518 [4.387, 4.655] (R=1.000) | ” | –0.023 [–0.059, 0.012] (R=1.000) | ” | –0.001 [–0.047, 0.044] (R=1.000) |
| 8 | ” | 0.496 [0.466, 0.528] (R=1.000) | **0.7** | 0.694 [0.678, 0.710] (R=1.000) | ” | 4.611 [4.442, 4.788] (R=1.000) | ” | –0.027 [–0.063, 0.009] (R=1.000) | ” | –0.028 [–0.071, 0.014] (R=1.000) |
| 9 | ” | 0.503 [0.471, 0.535] (R=1.001) | **0.9** | 0.893 [0.880, 0.907] (R=1.000) | ” | 4.511 [4.326, 4.710] (R=1.001) | ” | 0.029 [–0.005, 0.063] (R=1.000) | ” | 0.010 [–0.030, 0.048] (R=1.000) |
| 10 | ” | 0.518 [0.464, 0.574] (R=1.000) | 0.5 | 0.499 [0.459, 0.541] (R=1.000) | **1.0** | 0.976 [0.937, 1.018] (R=1.001) | ” | 0.009 [–0.013, 0.030] (R=1.000) | ” | 0.007 [–0.016, 0.029] (R=1.000) |
| 11 | ” | 0.502 [0.464, 0.541] (R=1.002) | ” | 0.525 [0.496, 0.554] (R=1.000) | **1.5** | 1.512 [1.463, 1.563] (R=1.002) | ” | 0.004 [–0.019, 0.027] (R=1.000) | ” | 0.001 [–0.026, 0.026] (R=1.000) |
| 12 | ” | 0.517 [0.488, 0.546] (R=1.000) | ” | 0.509 [0.490, 0.529] (R=1.000) | **3.0** | 2.952 [2.866, 3.036] (R=1.001) | ” | –0.009 [–0.039, 0.021] (R=1.000) | ” | 0.017 [–0.020, 0.055] (R=1.000) |
| 13 | ” | 0.482 [0.453, 0.513] (R=1.001) | ” | 0.515 [0.497, 0.532] (R=1.000) | **6.0** | 6.057 [5.827, 6.293] (R=1.002) | ” | **† 0.048 [0.007, 0.089] (R=1.000)** | ” | 0.040 [–0.010, 0.089] (R=1.000) |
| 14 | ” | 0.525 [0.491, 0.559] (R=1.000) | ” | 0.496 [0.479, 0.513] (R=1.000) | **7.5** | 7.284 [6.979, 7.604] (R=1.000) | ” | –0.005 [–0.050, 0.041] (R=1.000) | ” | 0.005 [–0.050, 0.059] (R=1.001) |
| 15 | ” | 0.516 [0.488, 0.546] (R=1.000) | ” | 0.508 [0.491, 0.525] (R=1.000) | 4.5 | 4.383 [4.245, 4.525] (R=1.001) | **–0.2** | –0.198 [–0.236, –0.161] (R=1.001) | ” | 0.023 [–0.022, 0.068] (R=1.000) |
| 16 | ” | 0.503 [0.476, 0.532] (R=1.000) | ” | 0.501 [0.484, 0.518] (R=1.000) | ” | 4.461 [4.323, 4.603] (R=1.000) | **–0.1** | –0.087 [–0.124, –0.050] (R=1.000) | ” | 0.046 [–0.000, 0.092] (R=1.000) |
| 17 | ” | 0.501 [0.474, 0.529] (R=1.001) | ” | 0.500 [0.483, 0.517] (R=1.000) | ” | 4.471 [4.333, 4.615] (R=1.001) | **0.1** | 0.073 [0.036, 0.109] (R=1.000) | ” | –0.020 [–0.066, 0.026] (R=1.000) |
| 18 | ” | 0.474 [0.448, 0.501] (R=1.000) | ” | 0.509 [0.492, 0.526] (R=1.001) | ” | 4.625 [4.474, 4.782] (R=1.000) | **0.2** | 0.219 [0.182, 0.256] (R=1.000) | ” | 0.007 [–0.041, 0.054] (R=1.000) |
| 19 | ” | 0.503 [0.480, 0.526] (R=1.001) | ” | 0.504 [0.490, 0.520] (R=1.001) | ” | 4.558 [4.434, 4.689] (R=1.001) | 0 | –0.005 [–0.039, 0.030] (R=1.000) | **–0.5** | –0.536 [–0.587, –0.485] (R=1.001) |
| 20 | ” | 0.486 [0.461, 0.513] (R=1.000) | ” | 0.503 [0.487, 0.520] (R=1.000) | ” | 4.481 [4.347, 4.622] (R=1.000) | ” | 0.004 [–0.032, 0.038] (R=1.000) | **–0.25** | –0.229 [–0.276, –0.182] (R=1.000) |
| 21 | ” | 0.519 [0.489, 0.550] (R=1.000) | ” | 0.497 [0.479, 0.514] (R=1.000) | ” | 4.461 [4.315, 4.613] (R=1.000) | ” | –0.010 [–0.047, 0.027] (R=1.000) | **0.25** | 0.237 [0.193, 0.284] (R=1.000) |
| 22 | ” | 0.495 [0.462, 0.529] (R=1.000) | ” | 0.497 [0.478, 0.516] (R=1.000) | ” | 4.607 [4.433, 4.791] (R=1.000) | ” | –0.023 [–0.063, 0.017] (R=1.000) | **0.5** | 0.482 [0.434, 0.528] (R=1.000) |

**Tables 2-11:** Correlations between individual subject parameter values and individual behavioural measures reported by Ersche et al. (2011). Separate tables are presented for the 5 parameters of the winning model. Tables 2-6 are separated by drug condition. Data in Tables 7-11 are collapsed across drug conditions. All *p* values are uncorrected. Plac = placebo. Ami = amisulpride. Prami = pramipexole. N = number. Persev = perseverative/perseveration. ns = not significant.

**Table 2:**

| **Stimulus Stickiness, *τ^stim^*** | | | | | | | | | |
| --- | --- | --- | --- | --- | --- | --- | --- | --- | --- |
|  | **HC** | | | **SUD** | | | **OCD** | | |
| **Behavioural**  **Measure** | **Plac** | **Ami** | **Prami** | **Plac** | **Ami** | **Prami** | **Plac** | **Ami** | **Prami** |
| N Trials per Sequence | ns | ns | ns | ns | ns | ns | r = .479, p = .044 | r = .510, p = .031 | r = .585, p = .011 |
| N Persev Errors | r = .587, p = .01 | ns | ns | ns | ns | ns | ns | ns | ns |
| N Sequences on Which Criterion for Persev | ns | ns | ns | ns | ns | ns | ns | ns | ns |
| Persev Error Rate | r = .672,  p = .002 | ns | ns | ns | ns | ns | ns | ns | ns |
| N Spontaneous Errors | ns | ns | ns | ns | ns | ns | r = .499,  p = .035 | ns | r = .558, p = .016 |
| N Probabilistic Switches | ns | ns | ns | ns | ns | ns | ns | ns | ns |

**Table 3:**

| **Reward Rate, *α^rew^*** | | | | | | | | | |
| --- | --- | --- | --- | --- | --- | --- | --- | --- | --- |
|  | **HC** | | | **SUD** | | | **OCD** | | |
| **Behavioural**  **Measure** | **Plac** | **Ami** | **Prami** | **Plac** | **Ami** | **Prami** | **Plac** | **Ami** | **Prami** |
| N Trials per Sequence | ns | ns | ns | r = -.571  p = .017 | ns | ns | ns | ns | r = -.547, p = .019 |
| N Persev Errors | ns | ns | ns | ns | ns | ns | ns | ns | ns |
| N Sequences on Which Criterion for Persev | ns | ns | ns | r = .611  p = .009 | ns | ns | ns | ns | ns |
| Persev Error Rate | ns | ns | r = -.546, p = .019 | ns | ns | ns | ns | ns | r = -.48, p = .044 |
| N Spontaneous Errors | ns | ns | ns | r = -.752  p = 4.92x10^-4^ | ns | ns | ns | ns | r = -.510, p = .031 |
| N Probabilistic Switches | ns | ns | ns | ns | ns | r = -.564, p = .018 | ns | ns | ns |

**Table 4:**

| **Punishment (Nonreward) Rate, *α^pun^*** | | | | | | | | | |
| --- | --- | --- | --- | --- | --- | --- | --- | --- | --- |
|  | **HC** | | | **SUD** | | | **OCD** | | |
| **Behavioural**  **Measure** | **Plac** | **Ami** | **Prami** | **Plac** | **Ami** | **Prami** | **Plac** | **Ami** | **Prami** |
| N Trials per Sequence | r = .664,  p = .003 | r = .478,  p = .045 | ns | ns | ns | ns | r = .661,  p = .003 | ns | r = .604,  p = .008 |
| N Persev Errors | ns | r = -.635,  p = .005 | r = -.632,  p = .005 | ns | r = -.486, p = .048 | r = -.497,  p = .043 | r = -.732,  p = .001 | r = -.557,  p = .016 | r = -.512,  p = .03 |
| N Sequences on Which Criterion for Persev | r = -.617,  p = .006 | r = -.862,  p = 4x10^-6^ | r = -.823,  p = 2.80x10^-5^ | r = -.502,  p = .04 | r = -.534, p = .027 | ns | r = -.888,  p = 8.9x10^-7^ | r = -.784,  p = 1.17x10^-4^ | r = -.841,  p = 1.20x10^-5^ |
| Persev Error Rate | ns | ns | ns | ns | ns | ns | r = .520  p = .027 | ns | ns |
| N Spontaneous Errors | r = .592,  p = .01 | r = .473,  p = .047 | r = .705,  p = .001 | ns | ns | ns | r = .592,  p = .01 | r = .513,  p = .029 | r = .529,  p = .024 |
| N Probabilistic Switches | r = .748,  p = 3.58x10^-4^ | r = .886,  p = 1x10^-6^ | r = .849,  p = 8x10^-6^ | r = .815,  p = 6.7x10^-5^ | r = .884,  p = 2x10^-6^ | r = .702,  p = .002 | r = .858,  p = 5x10^-6^ | r = .726,  p = .001 | r = .727,  p = .001 |

**Table 5:**

| **Side Stickiness, *τ^loc^*** | | | | | | | | | |
| --- | --- | --- | --- | --- | --- | --- | --- | --- | --- |
|  | **HC** | | | **SUD** | | | **OCD** | | |
| **Behavioural**  **Measure** | **Plac** | **Ami** | **Prami** | **Plac** | **Ami** | **Prami** | **Plac** | **Ami** | **Prami** |
| N Trials per Sequence | r = -.566,  p = .014 | ns | ns | ns | ns | ns | ns | ns | ns |
| N Persev Errors | ns | r = .773,  p = 1.69x10^-4^ | r = .499,  p = .035 | ns | ns | ns | ns | ns | ns |
| N Sequences on Which Criterion for Persev | ns | r = .554,  p = .017 | ns | ns | ns | ns | ns | ns | ns |
| Persev Error Rate | ns | r = .473,  p = .048 | ns | ns | ns | ns | ns | ns | ns |
| N Spontaneous Errors | r = -.51,  p = .031 | ns | r = -.585,  p = .011 | ns | ns | ns | ns | ns | ns |
| N Probabilistic Switches | r = -.489,  p = .04 | r = -.614,  p = .007 | ns | ns | ns | ns | ns | ns | ns |

**Table 6:**

| **Reinforcement Sensitivity, *τ^reinf^*** | | | | | | | | | |
| --- | --- | --- | --- | --- | --- | --- | --- | --- | --- |
|  | **HC** | | | **SUD** | | | **OCD** | | |
| **Behavioural**  **Measure** | **Plac** | **Ami** | **Prami** | **Plac** | **Ami** | **Prami** | **Plac** | **Ami** | **Prami** |
| N Trials per Sequence | r = -.62,  p = .006 | r = -.66,  p = .003 | r = -.638,  p = .004 | r = -.49,  p = .046 | r = -.585,  p = 014 | r = -.708,  p = .001 | r = -.707,  p = .001 | r = -.641,  p = .004 | r = -.632,  p = .005 |
| N Persev Errors | ns | r = .546,  p = .019 | r = .543,  p = .02 | ns | ns | r = .728,  p = .001 | ns | ns | ns |
| N Sequences on Which Criterion for Persev | r = .714,  p = .001 | r =.684,  p = .002 | r = .756,  p = 2.88x10^-4^ | r = .538,  p = .026 | r = .659,  p = .004 | r = .745,  p = .001 | r = .619,  p = .006 | ns | ns |
| Persev Error Rate | r = -.53,  p = .024 | ns | ns | ns | r = -.552,  p = .022 | ns | r = -.578,  p = .012 | ns | ns |
| N Spontaneous Errors | r = -.586,  p = .011 | r = -.68,  p = .002 | r = -.807,  p = 5.10x10^-5^ | ns | r = -.681,  p = .003 | r = -.77,  p = 2.96x10^-4^ | r = -.724,  p = .001 | r = -.697,  p = .001 | r = -.621,  p = .006 |
| N Probabilistic Switches | r = -.541,  p = .02 | r = -.551,  p = .018 | r = -.533,  p = .023 | ns | ns | ns |  | ns | ns |

**Table 7:**

| **Stimulus Stickiness, *τ^stim^*, Drugs Averaged** | | | |
| --- | --- | --- | --- |
| **Behavioural**  **Measure** | **HC** | **SUD** | **OCD** |
| N Trials per Sequence | ns | ns | r = .624, p = .006 |
| N Persev Errors | ns | ns | ns |
| N Sequences on Which Criterion for Persev | ns | ns | ns |
| Persev Error Rate | ns | ns | ns |
| N Spontaneous Errors | ns | ns | r = .548, p = .018 |
| N Probabilistic Switches | ns | ns | ns |

**Table 8:**

| **Reward Rate, *α^rew^*, Drugs Averaged** | | | |
| --- | --- | --- | --- |
| **Behavioural**  **Measure** | **HC** | **SUD** | **OCD** |
| N Trials per Sequence | ns | ns | ns |
| N Persev Errors | ns | ns | ns |
| N Sequences on Which Criterion for Persev | ns | ns | ns |
| Persev Error Rate | r = -.494, p = .037 | ns | ns |
| N Spontaneous Errors | ns | ns | ns |
| N Probabilistic Switches | ns | ns | ns |

**Table 9:**

| **Punishment Rate, *α^pun^*, Drugs Averaged** | | | |
| --- | --- | --- | --- |
| **Behavioural**  **Measure** | **HC** | **SUD** | **OCD** |
| N Trials per Sequence | r = .537, p = .022 | ns | r = .641, p = .004 |
| N Persev Errors | r = -.627, p = .005 | r = -.631, p = .007 | r = -.712, p = .001 |
| N Sequences on Which Criterion for Persev | r = -.860, p = 5 x 10^-6^ | r = -.577, p = .015 | r = -.927, p = 3.33 x 10^-8^ |
| Persev Error Rate | ns | ns | r = .504, p = .033 |
| N Spontaneous Errors | r = .738, p = 4.71 x 10^-4^ | ns | r = .6, p = .009 |
| N Probabilistic Switches | r = .897, p = 4.70 x 10^-7^ | r = .889, p = 2 x 10^-6^ | r = .913, p = 1.26 x 10^-7^ |

**Table 10:**

| **Side Stickiness, *τ^loc^*, Drugs Averaged** | | | |
| --- | --- | --- | --- |
| **Behavioural**  **Measure** | **HC** | **SUD** | **OCD** |
| N Trials per Sequence | ns | ns | ns |
| N Persev Errors | r = .622, p = .006 | ns | ns |
| N Sequences on Which Criterion for Persev | ns | ns | ns |
| Persev Error Rate | ns | ns | ns |
| N Spontaneous Errors | r = -.55, p = .018 | ns | ns |
| N Probabilistic Switches | r = -.569, p = .014 | ns | ns |

**Table 11:**

| **Reinforcement Sensitivity, *τ^reinf^*, Drugs Averaged** | | | |
| --- | --- | --- | --- |
| **Behavioural**  **Measure** | **HC** | **SUD** | **OCD** |
| N Trials per Sequence | r = -.716, p = .001 | r = -.67, p = .003 | r = -.775, p = 1.58 x 10^-4^ |
| N Persev Errors | r = .476, p = .046 | r = .589, p = .013 | ns |
| N Sequences on Which Criterion for Persev | r = .776, p = 1.53 x 10^-4^ | r = .739, p = .001 | r = .572, p = .013 |
| Persev Error Rate | ns | ns | r = -.532, p = .023 |
| N Spontaneous Errors | r = -.84, p = 1.3 x 10^-5^ | r = -.8, p = 1.17 x 10^-4^ | r = -.743, p = 4.11 x 10^-4^ |
| N Probabilistic Switches | r = -.577, p = .012 | ns | r = -.487, p = .04 |

**Scripts and associated files:**

Core associated files:

| Filename | Description |
| --- | --- |
| fixdata_jk.py | Preprocessor to generate *jk_all_data.csv* from multiple input files. |
| jk_all_data.csv | Composite data file. |
| slurm_submit_kanen.py | SLURM-based launch script for our cluster. Launches *bayesian_reversals_jk.R* for all required models. |
| bayesian_reversals_jk.R | Master coordinating R script. |
| reversals_*.stan | Model files (with numerical names, representing the order of creation, and differing from the numbering used in the manuscript). The mapping (from code to manuscript) is as follows: 1→4a, 2→4c, 3→4b, 4→3, 5→2, 6→5, 7→1. Model files with “s” appended to their name also capture per-subject parameters in the “generated quantities” block. |
| data.stan | Stan inclusion file describing the data structure used by all models. |
| constants.stan | Stan inclusion file describing fixed constants (including priors) used by all models. |
| sim_from_model.py | Python 3.6+ program to simulate behavioural data in the probabilistic reversal learning task of Ersche et al. (2011) from parameters estimated from Model 4c, the winning model |
| PRL_simulation_analysis_final.m | Matlab script to analyse synthetic data from the winning model. |
| analyse_parameter_recovery.R | R script to analyse synthetic data from the parameter recovery demonstration. |

Support files:

| Filename | Description |
| --- | --- |
| commonfunc.stan | Stan inclusion file (common functions, including to ease working with the bridgesampling package). |
| make_commonfunc_stan.py | Python script to generate *commonfunc.stan*. |
| listassign.R | R helper file (list assignment). |
| listfunc.R | R helper file (list-related functions). |
| miscfile.R | R helper file (file-related functions). |
| miscmath.R | R helper file (mathematical functions). |
| miscstat.R | R helper file (statistical functions). |
| rpm.R | R helper file (randomized probability matching). |
| stanfunc.R | R helper file (Stan functions). |
